# The Plant Immunity Regulating F-Box Protein *CPR1* Supports Plastid Function in Absence of Pathogens

**DOI:** 10.3389/fpls.2017.01650

**Published:** 2017-09-22

**Authors:** Christiane Hedtmann, Wei Guo, Elena Reifschneider, Isabelle Heiber, Heiko Hiltscher, Jörn van Buer, Aiko Barsch, Karsten Niehaus, Beth Rowan, Tobias Lortzing, Anke Steppuhn, Margarete Baier

**Affiliations:** ^1^Plant Physiology, Dahlem Centre of Plant Sciences, Free University of Berlin Berlin, Germany; ^2^Plant Physiology and Biochemistry, Bielefeld University Bielefeld, Germany; ^3^Plant Sciences, Heinrich Heine University of Düsseldorf Düsseldorf, Germany; ^4^Proteom- und Metabolomforschung, Bielefeld University Bielefeld, Germany; ^5^Department of Molecular Biology, Max Planck Institute for Developmental Biology Tübingen, Germany; ^6^Department of Molecular Ecology, Free University of Berlin Berlin, Germany

**Keywords:** Arabidopsis, chloroplast, CPR1, reactive oxygen species, redox imbalanced mutant, signaling

## Abstract

The *redox imbalanced 6* mutant (*rimb6)* of *Arabidopsis thaliana* was isolated in a genetic screening approach for mutants with defects in chloroplast-to-nucleus redox signaling. It has an atypically low activation status of the 2-Cys peroxiredoxin-A promoter in the seedling stage. *rimb6* shows wildtype-like germination, seedling development and greening, but slower growth and reduced biomass in the rosette stage. Mapping of the casual mutation revealed that *rimb6* carries a single nucleotide polymorphism in the gene encoding *CONSTITUTIVE EXPRESSER OF PATHOGENESIS RELATED (PR) GENES 1, CPR1* (At4g12560), leading to a premature stop codon. CPR1 is known as a repressor of pathogen signaling and regulator of microtubule organization. Allelism of *rimb6* and *cpr1* revealed a function of CPR1 in chloroplast stress protection. Expression studies in pathogen signaling mutants demonstrated that CPR1-mediated activation of genes for photosynthesis and chloroplast antioxidant protection is, in contrast to activation of pathogen responses, regulated independently from PAD4-controlled salicylic acid (SA) accumulation. We conclude that the support of plastid function is a basic, SA-independent function of CPR1.

## Introduction

Plants are prone to generating reactive oxygen species (ROS). Besides ROS-formation in peroxisomes and at the plasma membrane (Auh and Murphy, 1995; Deliro et al., 1996), the photosynthetic electron transport chain is one of the main ROS sources in plant cells (Foyer et al., 1994). ROS, like singlet oxygen (${\text{O}}_{2}^{1}$), superoxide anions (${\text{O}}_{2}^{-}$) and H_2_O_2_, are generated in plastids. If the ROS-levels are insufficiently controlled, membranes, metabolites and proteins get damaged (Baier and Dietz, 1999a). Even at low doses, ROS also initiate bouquets of signaling cascades (Vranova et al., 2002; Pfannschmidt, 2003; Baier and Dietz, 2005; Gadjev et al., 2006), induce biosynthesis of the stress hormone salicylic acid (SA) (Ishiga et al., 2012; Maruta et al., 2012) and activate systemic immune signaling (Miller et al., 2009; Szechynska-Hebda et al., 2010).

A network of antioxidant enzymes and low-molecular-weight antioxidants counteracts accumulation of ROS (Foyer et al., 1994; Asada, 2000). 2-Cys peroxiredoxin A (2CPA) is an evolutionarily ancient and abundant peroxidase in the plastid antioxidant system (PAS) (Baier and Dietz, 1996; König et al., 2002). It is highly expressed in young mesophyll cells and responds to photosynthetic redox signals (Baier et al., 2004) via the AP2-type transcription factor RAP2.4a (Shaikhali et al., 2008) and the transcription factor-interacting protein RCD1 (Hiltscher et al., 2014). In the PAS, 2CPA is accompanied by other peroxidases and superoxide dismutases and low molecular weight antioxidants (Foyer et al., 1994; Asada, 2000; Baier et al., 2010).

To dissect the signaling pathways regulating expression of PAS enzymes, we isolated the *redox imbalanced* (*rimb*) mutants in a genetic screening approach after chemical mutagenesis of a reporter gene line expressing luciferase under the control of the *2CPA* promoter. All *rimb*-mutants show low *2CPA* promoter activity, but increased oxidation of chloroplast proteins (Heiber et al., 2007). Germination, early seedling development and greening are unaffected. Besides *2CPA*, expression of various other genes for chloroplast proteins is decreased (Heiber et al., 2007).

*Rimb6* is one of the mutants, that was isolated based on decreased *2CPA*-promoter activity at an age of 10 days (Heiber et al., 2007). In young leaves, the chloroplast ultrastructure is undistinguishable from wildtype (Heiber et al., 2007). More starch granules with high electron density are formed later and they resemble the starch granules produced in Arabidopsis leaves under carbohydrate excess conditions (Pena-Ahumada et al., 2006; Heiber et al., 2007). Scoring of the F_2_ population of the backcross of *rimb6* to its non-mutagenized parental reporter gene line T19-2 demonstrated that the mutation is inherited as a recessive trait (Heiber et al., 2007). Of all *rimb*-mutants, *rimb6* showed strongest oxidation of chloroplast proteins and strongest activation of extra-plastidic peroxidase activity and catalase in the young rosette stage. The low-molecular weight antioxidants ascorbate and glutathione accumulated in response to the insufficient expression of various PAS enzymes (Heiber et al., 2007).

Here, we show that the *rimb6* mutant carries its casual mutation in the gene encoding the *CONSTITUTIVE EXPRESSER OF PATHOGENESIS RELATED (PR) GENES 1* (*CPR1*, At4g12560) and propose that activation of the immune responses in *cpr1* mutants is supported by insufficient plastid antioxidant protection.

## Materials and methods

### Plant material and growth conditions

*Arabidopsis thaliana* lines were grown in a 10 h light/14 h dark regime on soil as described in Juszczak et al. (2012). For the experiments depicted in **Figures 7**–**9**, the plants were grown in a 10 h light/14 h dark regime on 50% MS-medium supplemented with 0.5% sucrose as optimized for growth and expression of genes for chloroplast proteins in Heiber et al. (2014). For induction of flowering, 4 week old plants were transferred to a day/night regime of 16 h light/8 h dark.

*rimb6* is an ethyl methanesulfonate mutant of T19-2 (Heiber et al., 2007), which expresses luciferase under control of the 2CPA promoter (Baier et al., 2004). The T-DNA insertion line SALK_111420 (Alonso et al., 2003) and the pathogen signaling mutants were obtained from NASC (http://www.arabidopsis.info). Homozygosity of the T-DNA insertion was confirmed by PCR (Primers Supplementary Table 1). Plant lines were crossed by transferring the pollen of the father plant on the stigma of an emasculated mother plant. The T_1_ and T_2_ offspring was tested for presence of the T-DNA by PCR. For differentiation of wildtype and *cpr1-4* alleles, cDNA or genomic DNA was amplified with the primers CPR1-4 CAPS-F TTGATCTTGCCTTGGAAGAG and CPR1-4 CAPS-R ACAAGGCTACTCACAACGAG by PCR (30 cycles: 30 s 94°C, 30 s 56°C and 30 s 72°C) and digested with Fok-I, which cuts the 391 bp PCR product for the wildtype allele into 233 and 158 bp fragments and leaves the PCR product for the *cpr1-4* allel intact. 2% (w/v) agarose gels were run for distinguishing between the 391 bp mutant and the 233 bp wildtype fragments.

For HL treatment, the plants were exposed to 800 μmol quanta m^−2^ s^−1^ for 4 h after 1 h at normal light intensity. H_2_O_2_ was applied by floating Arabidopsis seedlings, which were germinated on MS-medium supplemented with 0.5% sucrose (Heiber et al., 2007), in liquid MS-medium supplemented with 10 μM H_2_O_2_ and 0.5% (w/v) sucrose. H_2_O_2_ was added 1 h after onset of light. For the controls and the HL-treatments depicted in **Figure 8**, the plants were floated for the same time on MS-medium supplemented only with 0.5% sucrose.

### Scanning electron microscopy

The surfaces of mature leaves of 6-week-old *rimb6* and T19-2 plants (grown under short-day conditions) were analyzed by scanning electron microscopy as described in Hiltscher et al. (2014).

### Mapping of the *RIMB6* locus

A *rimb6* mapping population was generated by crossing *rimb6* (in the Col-0 background of the line T19-2) (Heiber et al., 2007) to Ler. The F_2_ population was scored for low luciferase activity (Heiber et al., 2007) and the dwarf phenotype. For mapping with SSLP and CAPS markers (Jander et al., 2002), genomic DNA was extracted from 220 individual F_2_ seedlings. Marker information was taken from the Monsanto Arabidopsis Polymorphism Collection (Jander et al., 2002) and Bell and Ecker (1994).

For high-throughput sequencing, DNA was extracted from a pool of 78 and a pool of 130 plants from the F_2_ mapping population that exhibited reduced luciferase expression. For DNA extraction, 1 g of plant material for each pool was homogenized in 10 ml ice-cold nuclei extraction buffer [10 mM Tris-HCl (pH 9.5), 10 mM EDTA (pH 8.0), 100 mM KCl, 500 mM sucrose, 4 mM spermidine, 1 mM spermine and 0.1% (v/v) β-mercaptoethanol] and filtered through two layers of Miracloth (Calbiochem, MERCK, Germany). The samples were gently mixed in 2 ml lysis buffer (10% (v/v) Triton X-100 in nuclei extraction buffer) for 2 min on ice. Following a 10 min centrifugation at 2,000 g at 4°C, the sedimented nuclei were re-suspended in 500 μl CTAB buffer [100 mM Tris-HCl (pH 7.5), 0.7 M NaCl, 10 mM EDTA, 1% (v/v) β-mercaptoethanol and 1% (w/v) CTAB] and incubated for 30 min at 60°C. The samples were mixed by inversion for 5 min at room temperature following the addition of 350 μl chloroform-isoamyl alcohol (24:1). After 10 min centrifugation at 3,300 g, the DNA was precipitated in a 1:1 mixture of the upper phase and isopropanol by 3 min centrifugation at 15.700 g, washed in 75% (v/v) ethanol, dissolved in 50–100 μl DNase free water containing 10 μg/ml RNAseA and incubated for 20 min at 65°C prior to storage at −20°C.

The DNA quantity was determined with a Qubit fluorometer (Life Technologies, Germany). The quality was checked by electrophoresis on a 1.2% (w/v) agarose gel. An indexed paired-end DNA library was prepared for each sample according to Rowan et al. (2015) using 400 ng DNA for each pool and selecting for an insert size of 200–500 bp, and sequenced using a HiSeq2000 system (Illumina, San Diego, CA) with 2 × 100 bp reads.

The adapter sequences were clipped from raw reads, which were then filtered for quality, trimmed to a minimum length of 75 bp, before aligning to the *Arabidopsis thaliana* reference genome, allowing for a maximum of 10% mismatches and 7% gaps using the SHORE and GenomeMapper software programs (Ossowski et al., 2008; Schneeberger et al., 2009b). The alignments were corrected using paired-end information before polymorphism detection using SHORE. Finally, the allele frequencies of Col-0 and Ler-0 were determined using SHOREmap (Schneeberger et al., 2009b) and the boost function was applied to determine the final mapping interval. An annotated list of all SNPs was obtained using SHOREmap after filtering out the Ler polymorphisms to obtain a final list of candidate mutations.

For confirmation, the candidate gene was amplified with gene-specific primers (Supplementary Table 1) and OptiTaq polymerase (Roboklon, Germany) from the mutant and its parental line. Sanger sequencing (Sanger et al., 1977) of the PCR-products was performed by Eurofins MWG Operon (Ebersberg, Germany).

### qRT-PCR analysis

The shoots of 3–5 individual plants (per replicate) were pooled and immediately frozen in liquid nitrogen. Total RNA was extracted from 100 mg ground plant material using the GeneMatrix Universal RNA Purification Kit (Roboklon, Germany) with on-column treatment with RNase-free DNaseI (Fermentas, Germany). Oligo-d(T) primed cDNA was synthesized from 1 μg RNA with the High Capacity Reverse Transcription Kit (Applied Biosystems, Carlsbad, CA).

Real-time quantitative polymerase chain reactions (qRT-PCR) were performed as described in Hiltscher et al. (2014) or in a final volume of 20 μl containing 50 ng cDNA, 16 mM ammonium sulfate, 0.1 M Tris-HCl (pH 8.3), 0.01% (v/v) Tween-200, 2 mM MgCl_2_, 0.1 mM dNTP, 0.2 μl 10X SYBR Green (Sigma-Aldrich, Germany), 0.2 U OptiTaq Polymerase (Roboklon) and 0.3 μM gene-specific primers (Supplementary Table 1). Technical triplicates were run on a CFX96 Real-time System (BioRad, Hercules, CA) for at least three independent biological samples using 40 cycles 95°C/15 s, 60°C/30 s and 72°C/30 s after 5 min incubation at 95°C. Each assay included a standard curve of four serial dilution points of Col-0 cDNA (300 ng–300 pg) and a non-template control. Exon–intron border spanning primers were designed using QUANTPRIME (Arvidsson et al., 2008). Primer specificity was assessed by inspection of the melting curves after 40 cycles. The Cq values were determined using the regression model within the CFX Manager software v3.0 (BioRad, Hercules, CA) and analyzed with respect to amplification efficiency. The transcript levels were normalized on the geometric mean transcript level of the constitutively expressed genes *At3g18780* (*actin2*), *At5g15710* (*F-box*), and *At5g08290* (*YLS8*) (Czechowski et al., 2005).

### GC-MS analysis

100 mg plant material were immediately frozen in liquid nitrogen and lyophilized in 1 ml 80% methanol containing 10 μM ribitol (as an internal standard) and 0.5 g zirconia glass beads (Carl Roth, Germany) in a FastPrep™ Instrument (Qbiogene, Germany) using 45 cycles of 6.5 m s^−1^ followed by a 15 min incubation at 70°C with continuous shaking at 1,400 rpm. Following 20 min centrifugation at 13,000 g, the supernatant was dried in a nitrogen stream in glass vials. To extract metabolites, 50 μl of 20 mg/l methoxylamine hydrochloride (in pyridine) were added to the samples prior to incubation at 90 min at 37°C. After addition of 50 μl N-methyl-N-[trimethylsilyl] trifluoroacetamide they were incubated for 30 min at 37°C.

One μl of each sample was analyzed in a TraceGC gas chromatorgraph coupled to a PolarisQ ion trap mass spectrometer and an AS2000 auto sampler (Thermo Finnigan, Germany). Injection was performed at 250°C (splitness mode) and separation was achieved on a 30 m × 0.25 mm Equity-5 column with 0.25 μm coating (Supleco, Bellefonte, CA, USA) at an interface temperature of 250°C and an ion source temperature of 200°C in a constant flow of helium carrier gas of 1 ml min^−1^. Following 2 min constant heating at 80°C, the oven temperature was raised to 300°C with a speed of 3°C min^−1^. Mass spectra were recorded in a range of 50–550 m z^−1^. Metabolites were identified by comparison with the NIST98 (NIST, Gaithersburg, MD) database, pure standards and by using the Golm Metabolome Database (Kopka et al., 2005). Metabolite peak relative areas were quantified using the processing setup implemented in the Xcalibur software (Thermo Finnigan, Germany) and normalized to the peak area of the internal standard ribitol. Differences between T19-2 and *cpr1-4* were evaluated based on the *P*-value of pairwise *t*-tests (*P* < 0.1).

### Content and redox state of ascorbate and glutathione

The concentration of ascorbate and glutathione were determined in 8–12 biological replicates per genotype and treatment as described in Baier et al. (2000). The redox state was calculated by dividing the concentration determined for the oxidized form by the total concentration (oxidized plus reduced forms) for the same extract. The DHA/Asc and GSSG/2x GSH ratios were calculated by dividing the concentration of the oxidized form by the concentration determined for the reduced form.

### Quantification of phytohormone levels

For quantification of phytohormone levels approximately 150 mg frozen plant material was extracted in a FastPrep®-24 instrument (MP Biomedicals, USA) at 5 m s^−1^ for 60 s in 2 ml screw-cab-tubes containing 1.25 g of 2.8–3.3-mm-diameter Zirconox beads, (Mühlmeier Mahltechnik, Germany) and 1 ml ethylacetate, including 20 ng D4-SA (OlChemIm Ltd., Czech Republic) as an internal standard. After centrifugation (10 min at 15,000 g at 4°C), the supernatant was transferred to a 2 ml reaction tube and the pellet was extracted a second time with 1 ml pure ethylacetate. Supernatants from both extractions were combined and dried in a vacuum concentrator (concentrator 5301, Eppendorf, Germany). The residue was eluted in 400 μl of 70% methanol with 0.1% formic acid (v/v) at room temperature. The extract was centrifuged again for 10 min at 15,000 g and 4°C, and 200 μl of supernatant were transferred to HPLC-vials. Analysis was performed using a 7 μl injection into a UPLC-ESI-MS/MS (Synapt G2-S HDMS; Waters, Milford, USA). Chromatography was performed on a C18 column (Acquinity UPLC BEH-C18, ø 2.1 × 50 mm, with a particle size 1.7 μm) at 30°C and a flow rate of 250 μl/min. Water and methanol [each containing 0.1% formic acid (v/v)] were used as solvents in a gradient (methanol: 0 min: 30%, 1 min: 30%, 4.5 min: 90%, 8 min: 90%, 9 min: 30%) with a 3 min equilibration time between the runs. Tandem mass spectrometry was performed in negative ionization mode with parent/daughter ion selections of 137/93 (SA), 141/97 (D4-SA), 209/59 (JA), 215/59 (D6-JA), 263/153 (ABA), 269/153 (D6-ABA), 322/130 (JA-Ile), 328/130 (D6-JA-Ile). Peak areas of daughter ions were integrated using MassLynx™ Software (version 4.1, Waters) and the amount of phytohormones was calculated according to the internal standard.

### ROS staining

Histochemical staining for ${\text{O}}_{2}^{-}$ and H_2_O_2_ and semiquantitative analysis was performed with nitroblue tetrazolium (NBT) and 3,3-diaminobenzidine (DAB) as described in Juszczak et al. (2016).

### Data analysis

All quantitative data were subjected to statistical analysis using the two-tailed, pairwise *t*-test, ANOVA (Bonferroni/Tukey testing), pairwise *t*-test, X^2^-test or the pairwise Welch's test.

## Results

### *rimb6* mutation causes severe growth defects

**Figure 1:**
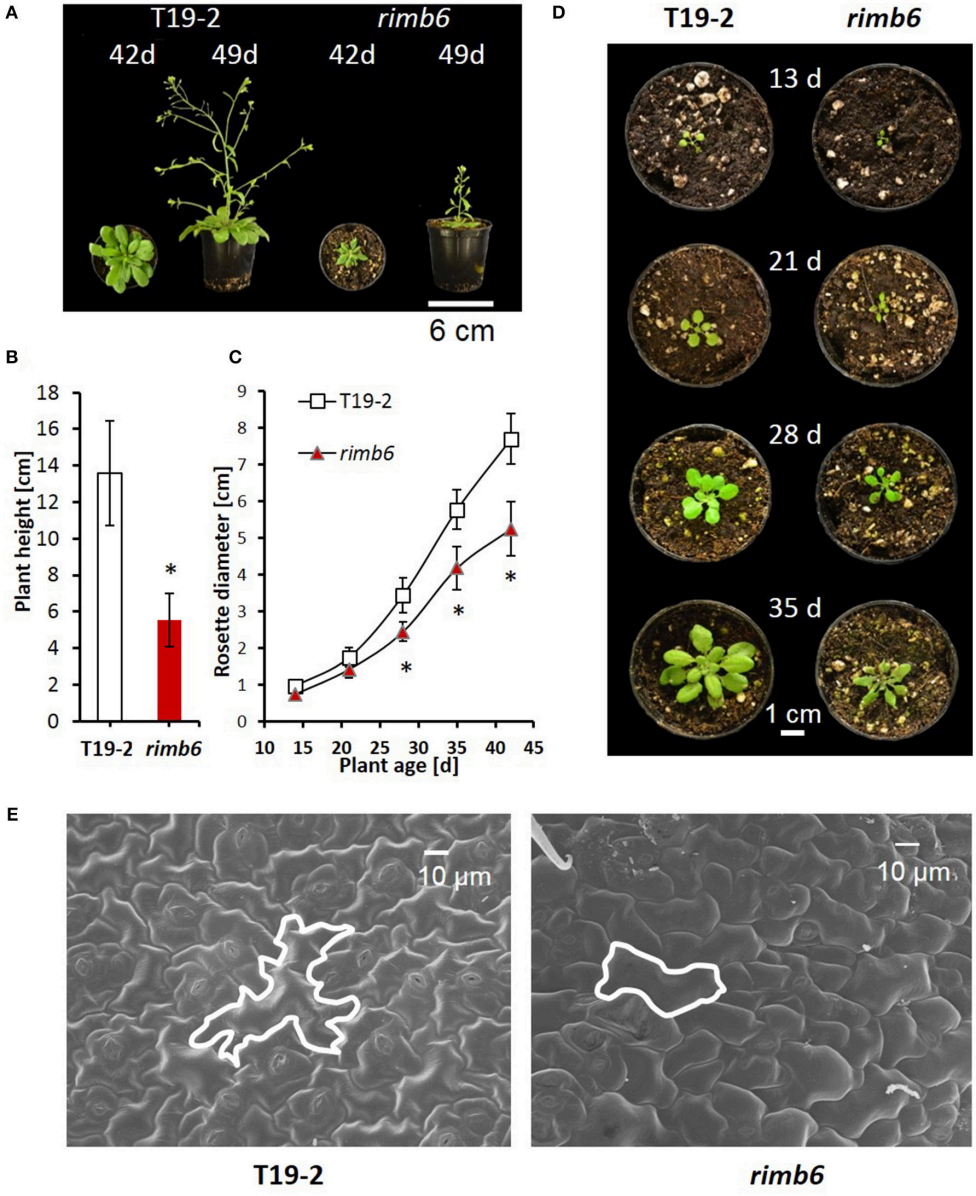
Phenotypic characteristics of the redox signaling mutant *rimb6* and its parental line T19-2. **(A)** Rosette morphology and shoot elongation of T19-2 and *rimb6* after 42 d and 49 d. **(B)** Plant height of T19-2 and *rimb6* (49 d) (*n* = 10; ^*^P < 0.01, pairwise *t*-test). **(C)** Rosette diameter in T19-2 and *rimb6* (*n* = 25; ^*^P < 0.01, pairwise *t*-test). **(D)** Representative images of *rimb6* and T19-2 during rosette development. **(E)** Laser scanning microscopy of upper leaf surfaces of *rimb6* and the parental line T19-2. One exemplary cell is outlined using a white line.

The growth habit of the *rimb6* mutant was indistinguishable from its parental line T19-2 during germination and at the seedling age. In the rosette stage, the leaves showed reduced expansion, the leaf margins were curled (Figure 1A, Supplementary Figure 1) and growth was slowed relative to T19-2 (Figures 1C,D). In mature leaves, the epidermal pavement cells were 30 ± 9% smaller and had fewer lobes (Figure 1E). However, *rimb6* did not show apoptotic mesophyll clefts as observed in *rimb1* (Hiltscher et al., 2014), which was isolated in the same screening approach (Heiber et al., 2007).

The onset of bolting was similar in *rimb6* and T19-2, when 4-week old plants were shifted to long-day conditions (14 h light/10 h dark), demonstrating that meristem reprogramming from vegetative to generative growth is unaffected in the mutant. The primary inflorescences of *rimb6* were shorter, had fewer branches and flowers than wildtype (wt) Col-0 or T19-2 (Figures 1A,B; Supplementary Figure 2). Many secondary shoots were released shortly after bolting, giving *rimb6* a bushy habitus (Supplementary Figure 1).

### Mapping of the *RIMB6* locus

The *RIMB6* locus was mapped by a combination of SSLP mapping (Jander et al., 2002) and high-throughput sequencing (Schneeberger et al., 2009a). Plants showing the *rimb6* dwarf phenotype were selected from the F_2_ population derived from a cross of *rimb6* and wildtype plants of the *Arabidopsis thaliana* accession *Landsberg erecta* (*Ler*). The *rimb6* mutation was localized in the phenotyped F_2_ population with simple sequence length polymorphism (SSLP) markers on chromosome IV between the markers NGA8 (Bell and Ecker, 1994) and CER46127 (Jander et al., 2002).

**Figure 2:**
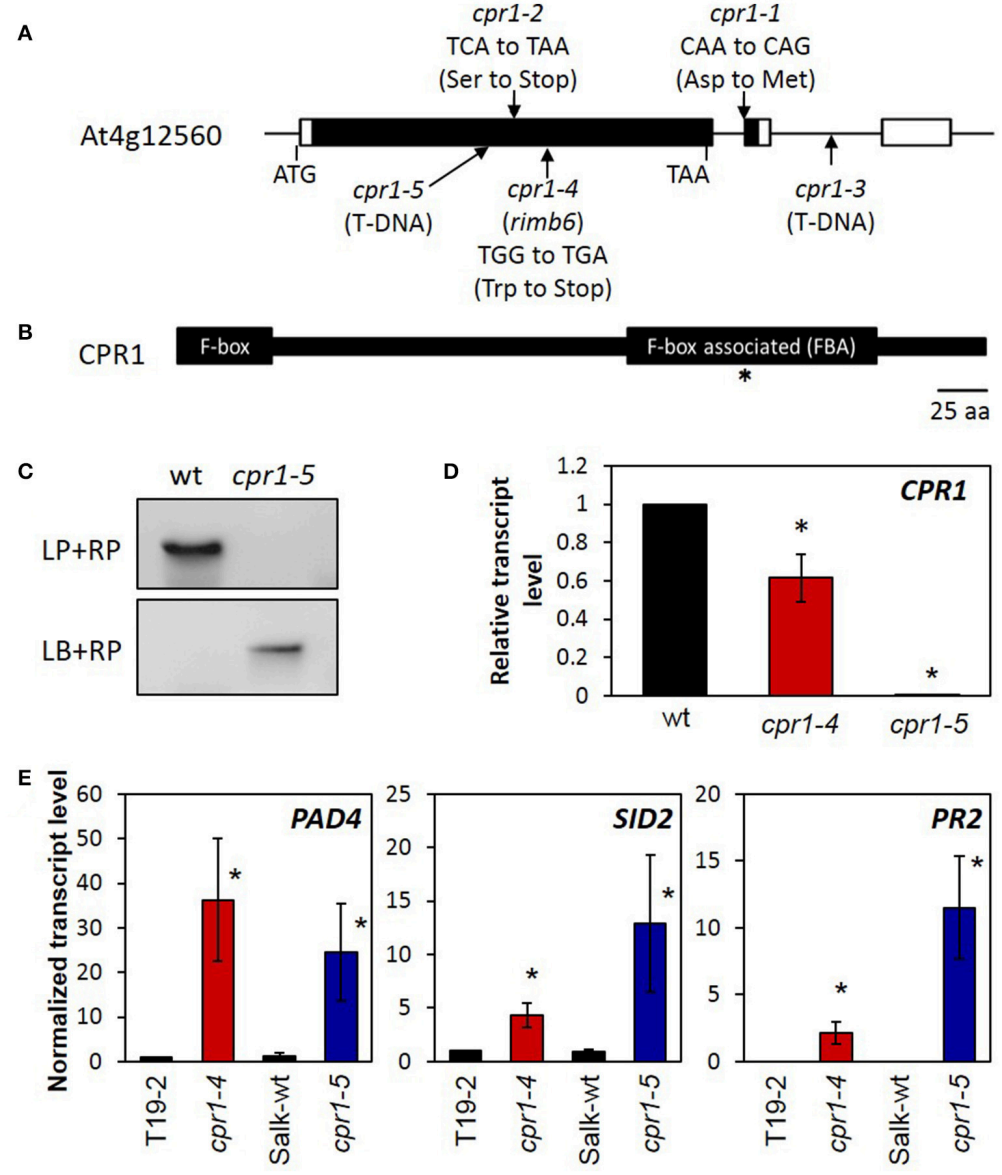
Mapping of *rimb6* to the *cpr1* locus. **(A)** The gene structure of the *CPR1*/*RIMB6* gene, (At4g12560.1). The black and white bars represent the coding and non-coding regions. The positions of the *rimb6* mutation, allelic point mutations, and a T-DNA insertion mutation are indicated. **(B)** The *RIMB6/CPR1* protein and its characteristic motifs. The F-box at the N-terminus and the F-box associated domain (FBA) are highlighted. The asterisk marks the mutation site in *rimb6/cpr1-4*. **(C)** Confirmation of the T-DNA insertion site of *cpr1-5*. Top panel: Amplification of *CPR1* with gene-specific primers (LP and RP) gives a product only with *wt* DNA. Bottom panel: PCR with a T-DNA specific and gene-specific primer only gives a product in the T-DNA insertion line *cpr1-5* confirming the T-DNA insertion. **(D)** Normalized transcript level of *CPR1* in 4-week-old soil-grown *cpr1-4* and *cpr1-5* relative to their corresponding genetic background lines. ^*^P < 0.1 (pairwise *t*-test for comparison of the mutants and their corresponding wildtypes). **(E)** Normalized transcript levels of defense response marker genes in 4-week-old soil-grown *cpr1* mutants and their corresponding controls. Bars represent the mean and standard errors of three biological replicates, each measured in triplicate by qRT-PCR and normalized to *At5g15710* (F-box) and *At5g08290 (YLS8)* transcript levels. Asterisks mark significant differences from T19-2 (^*^P < 0.1; ANOVA).

Illumina sequencing of two pools of F_2_ plants with low luciferase activity—one with 78 and one with 130 individuals—showed enrichment of Col-0 alleles (>90%) on chromosome IV between 7 and 9 Mb. After removing known Col/Ler single nucleotide polymorphisms (SNPs), 12 putative mutations remained (Supplementary Table 2). Three of the mutations were G/A substitutions and one was a T/A substitution. The other eight mutations were putative deletions. G/A substitutions result from chemically induced C/T substitutions and are most typical (>99%) for mutagenesis by ethyl methansulfonate (Greene et al., 2003). The G/A mutations at the positions 7,078,331 and 7,442,672 were non-synonymous and were investigated further as potential candidates. The mutation at position 7,442,672 was confirmed by sequence comparison of PCR products amplified from genomic DNA of *rimb6* and T19-2. The observed mutation was only found in *rimb6*-derived PCR products and absent from those amplified from the non-mutagenized line, T19-2. The other polymorphisms were found in both *rimb6* and T19-2 and could therefore not be causal for the phenotype. The candidate mutation changes a tryptophan codon, TGG, in the mRNA encoding the *CONSTITUTIVE EXPRESSOR OF PATHOGENESIS RESPONSE GENES 1* (*CPR1*) (At4g12560) into a stop codon, TGA, and terminates translation after 286 amino acids (Figure 2A). Wildtype CPR1 antagonizes effector triggered immunity (ETI) in the cytosol by mediating the proteasomal degradation of R-proteins, like SNC1 and RPS2 (Gou et al., 2009; Cheng et al., 2011). As one of its basic functions, besides antagonizing ETI induction, CPR1 controls microtubule arrangement (Han et al., 2015). The truncated CPR1 protein produced by the *rimb6* mutation lacks the C-terminal FBA domain (Figure 2B), which binds target proteins in an E3-ubiquitin ligase complex and guides R-proteins (Gou et al., 2009; Cheng et al., 2011) and other proteins (Wang et al., 2014) toward ubiquitinylation and degradation.

### Comparison of the *rimb6* mutant with a *cpr1*-T-DNA insertion line

The T-DNA insertion line SALK_111420 carries an insertion in the first exon of *CPR1* (Figure 2A). The homozygous line, which lacks detectable *CPR1* expression (Figure 2D), was phenotypically similar to *rimb6* (Supplementary Figure 2). Allelism of *rimb6* was confirmed by crossing *rimb6* (*cpr1-4*) with the T-DNA insertion line. *rimb6 x* SALK_111420 and SALK_111420 *x rimb6* F_1_ plants showed the mutant phenotype (Supplementary Figure 1), while reciprocal crosses of *rimb6* and its non-mutagenized parental line T19-2 did not. The *rimb6* mutant was renamed *cpr1-4* and the T-DNA insertion line SALK_111420 as *cpr1-5*.

qRT-PCR analysis with primers binding to the 3′-part of the At4g12560 transcript (= 5′-part of the oligo-dT-primed cDNA) (Supplementary Table 1) demonstrated that *cpr1* transcripts were present at lower levels in *cpr1-4* than the *CPR1* transcripts in *wt* plants (Figure 2D), indicating a feed-back of early translation termination on transcript stability.

### Expression of defense genes in the *cpr1-4* mutant

In wildtype plants, *CPR1* antagonizes induction of defense marker genes, e.g., *PHYTOALEXIN DEFICIENT 4 (PAD4), SALICYLIC ACID DEFICIENT 2 (SID2)* and *PATHOGENESIS RELATED GENE 2 (PR2)* (Gou et al., 2009), in the absence of pathogen stimuli by supporting degradation of R-proteins, such as SNC1 and RPS2 (Cheng et al., 2011; Gou et al., 2012). These genes were highly expressed in *cpr1-4 and cpr1-5* (Figure 2E), but barely detectable in T19-2 and the background line of *cpr1-5* (SALK-*wt*), demonstrating that both alleles constitutively activate defense gene expression like previously described for other *cpr1*-alleles (Gou et al., 2009).

### Complementation of the *cpr1-4* mutant with wildtype CPR1

**Figure 3:**
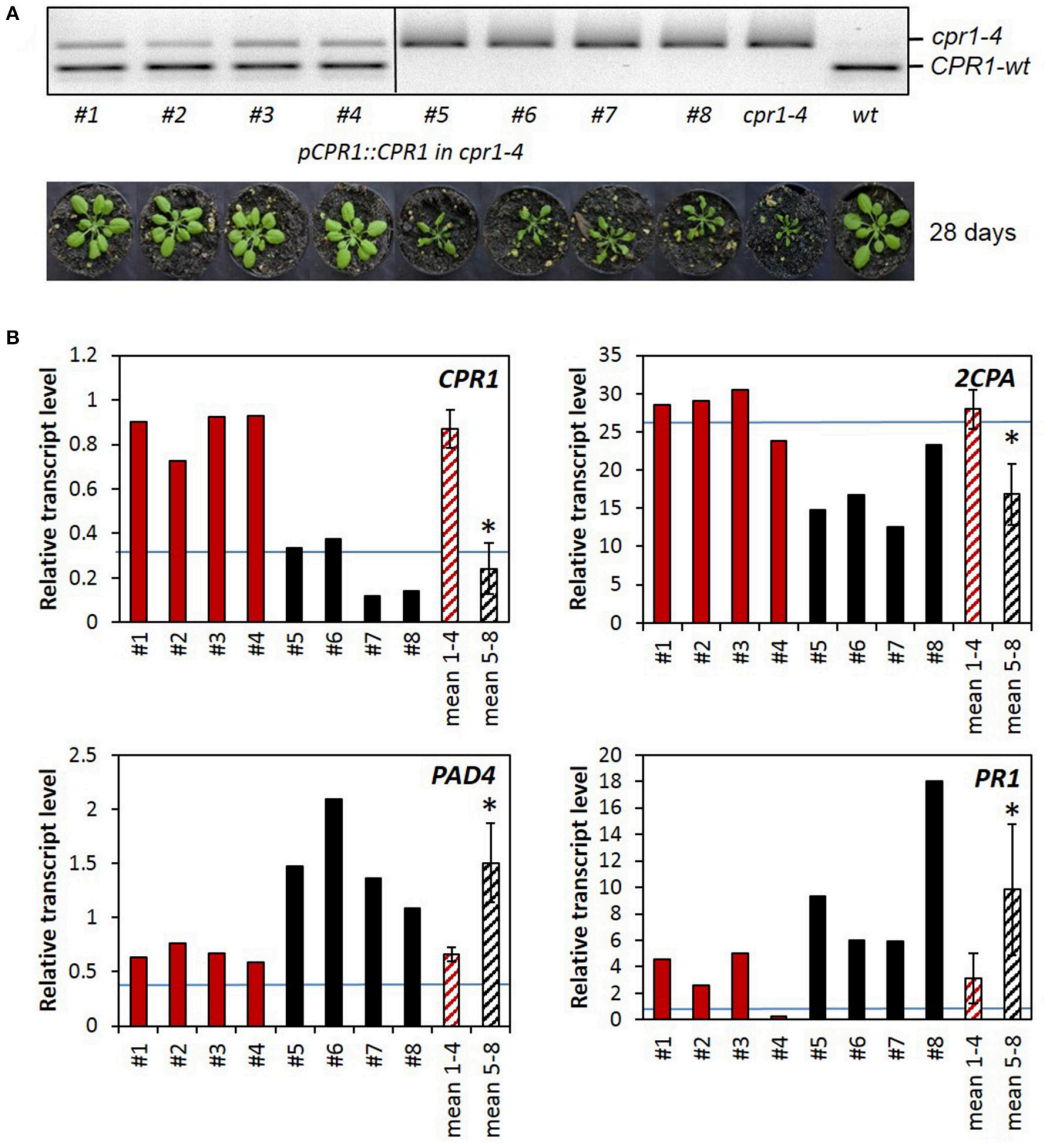
Complementation analysis. **(A)** CAPS-marker analysis and habitus of 4 *cpr1-4* lines expressing the *pCPR1::CPR1* complementation construct (#1-#4), 4 non complemented lines out of the same segregating T_2_ population (#5-#8), untransformed *cpr1-4* and Col-0 wildtype plants. The mutant allele gives a 391 bp fragment. From the wildtype allele, a 233 bp long FokI cleavage product is shown. **(B)** Relative transcript levels in 28-day-old plants: qRT-PCR was performed in triplicates with RNA isolated from single plants. For the two sets of 4 biological replicates, the means and standard deviations were calculated (mean 1-4 and mean 5-8) and the data sets were compared for significance of difference by pairwise *t*-test (*P* < 0.05; asterisks mark significant differences between the two mean values). The blue line marks the expression intensity of the respective gene in *Arabidopsis thaliana* wildtype plants.

To prove the causality between 2CPA miss-regulation and the *cpr1-4* mutation, the *cpr1-4* mutant was transformed with a construct expressing wildtype *CPR1* under control of the *CPR1* promoter (*pCPR1::CPR1* in *cpr1-4*). The segregating T_2_ population was screened with a CAPS marker (cleaved amplified polymorphic sequence) that amplifies a product where the wt CPR1 allele has a FokI cleavage site and *cpr1-4* does not (Figure 3A). Four T_2_ lines were selected, which express the wildtype allele, and four lines without the *pCPR1::CPR1* construct. Only the four T_2_-lines expressing the transgene showed wildtype growth and development, demonstrating complementation of the *cpr1-4* mutant phenotype.

The activity of the transgene was analyzed by qRT-PCR (Figure 3B). The *CPR1* transcript level was 3.6-fold higher in the *CPR1*-transgenic lines than in the *cpr1-4* mutant, indicating that approximately 2/3 of the *CPR1* transcripts in the *pCPR1::CPR1* transformants encode wildtype *CPR1*. In the complemented lines, the *2CPA* transcript levels were by average 1.6-fold higher than in *cpr1-4* and the *PAD4* transcript levels and the *PR1* transcript levels were significantly lower than in the *cpr1-4* mutant, and in the range of wildtype plants (blue lines in Figure 3B), demonstrating that CPR1 regulates *2CPA* and the two ETI genes *PAD4* and *PR1* inversely.

### The metabolome of *cpr1-4* shows a stress imprint

**Figure 4:**
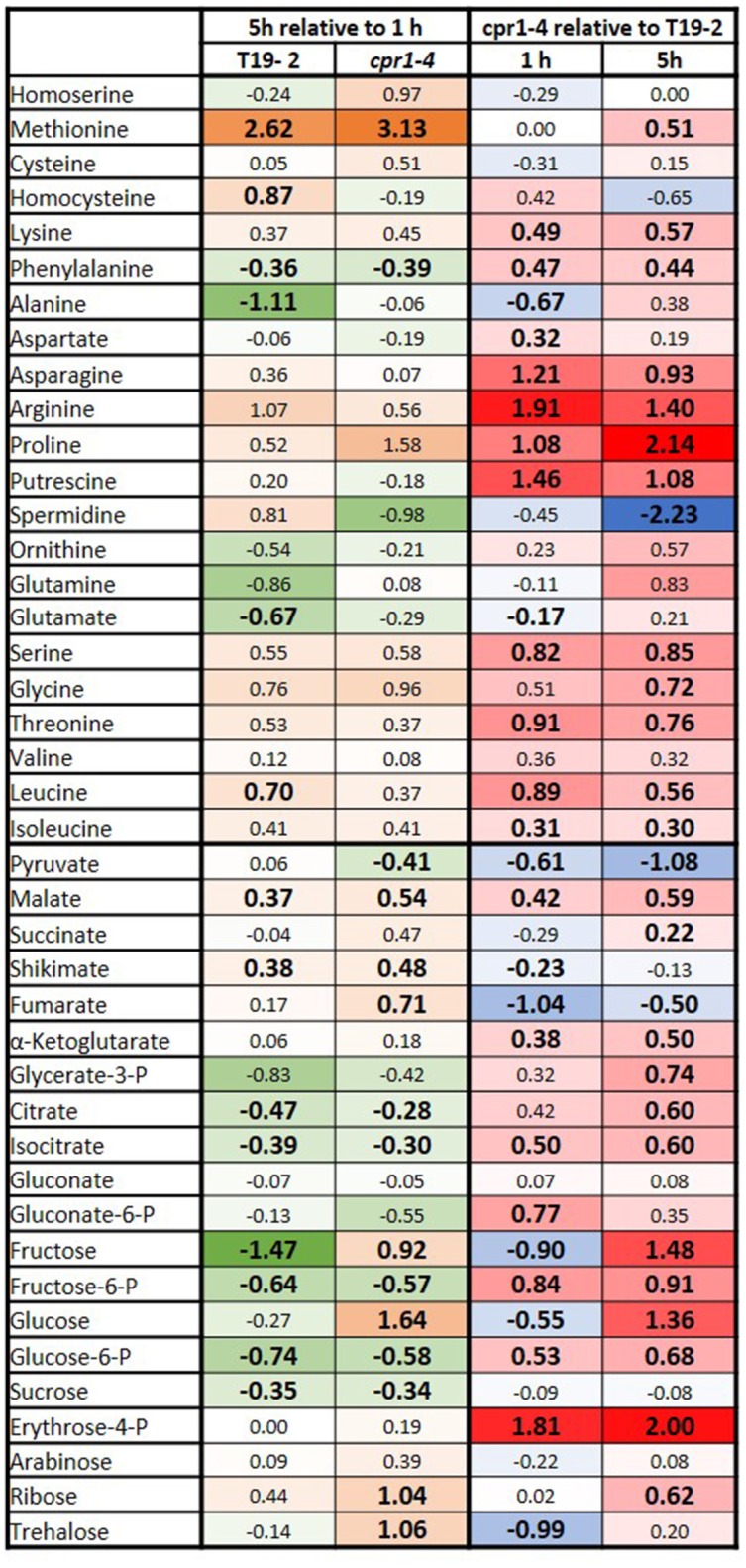
Heat-map depicting the log_2_ of the relative changes in carbohydrate and amino acid contents as determined by GC-MS in 4-week-old, soil-grown *cpr1-4* and T19-2. Left: The relative change between 5 and 1 h illumination in T19-2 and *cpr1-4*. The strongest decrease is presented in dark green (negative numbers), the strongest increase in orange (positive numbers). Right: The metabolite level in *cpr1-4* relative to T19-2. The highest relative level is shown in bright red (positive numbers), the lowest in blue (negative numbers). Unchanged metabolites are marked in white. Statistically significant regulation is presented in bold and slightly larger than the other numbers (*n* = 3 biological replicates; pairwise *t*-test, *P* < 0.1).

The relevance of CPR1 for chloroplast function tempted us to compare the carbohydrate and amino acid profiles in 28-day-old *cpr1-4* and T19-2 1 and 5 h after exposure to light by gas chromatography coupled to mass spectrometry (GS-MS) (Figure 4). The time-points address activation of light metabolism, which often diminishes the carbohydrate pools transiently, before carbohydrates and secondary photosynthates accumulate (Gibon et al., 2004; Zeeman et al., 2007; Figure 4, left).

The concentrations of glucose, fructose and pyruvate were lower in *cpr1-4* than in T19-2 1 h after onset of light (Figure 4, right). On the contrary, the concentrations of glucose-6-P, fructose-6-P and malate were all increased in *cpr1-4*, reflecting that the hexose energization status (hexose-P/hexose-ratio) and the reduction state of the malate/oxaloacetate system were both high. The levels of N-containing putrescine, aspartate, asparagine, threonine and leucine were also higher in *cpr1-4*.

After 5 h of light exposure, the glucose and fructose availabilities were restored to control levels in *cpr1-4* (Figure 4, right), but the pyruvate shortage remained and the glucose-6-P, fructose-6-P and serine levels were still increased. At this time point, the aspartate concentration had fallen to that of the control plants and the levels of the aspartate-derived amino acids threonine and asparagine were still increased. In addition to malate, citrate, isocitrate and α-ketoglutarate levels were enriched and stress metabolites such as ornithine, arginine, proline and glutamine and the pentose phosphate cycle intermediate erythrose-4-P accumulated. Taken together, these results show that the reduction status of metabolites, the hexose energization and the amination status were increased in *cpr1-4*.

### Regulation of stress hormones

**Figure 5:**
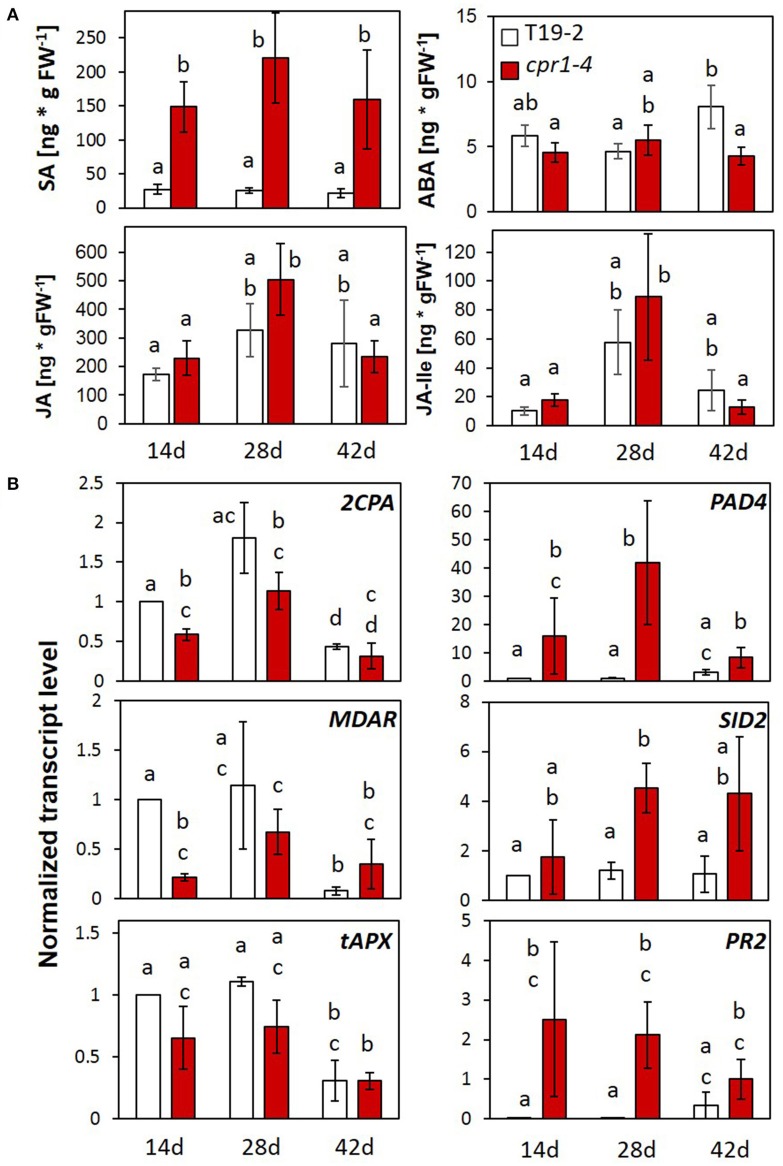
Impact of *CPR1* on the pathogen defense response and regulation of genes for chloroplast antioxidant enzymes during rosette development. **(A)** Total salicylic acid (SA), abscisic acid (ABA), jasmonate (JA) and jasmonate-isoleucine (JA-Ile) levels in 14-, 28-, and 42-day-old soil-grown *cpr1-4* and T19-2 plants (*n* = 6 biological replicates; Different letters show significance of difference; *P* < 0.05; pairwise Welch's test). **(B)** Normalized transcript levels of genes for chloroplast antioxidant enzymes, and defense response marker genes of *cpr1-4* and T19-2 plants during development [3 biological replicates, each measured in triplicate by qRT-PCR, normalized to *At5g15710* (*F-box*) and *At5g08290* (*YLS8*) transcript levels. *P* < 0.05; ANOVA].

The SA concentration was increased in *cpr1-4* (Figure 5A) as reported for *cpr1-2*/*cpr30* (Gou et al., 2009). Accumulation of SA at the youngest tested stage (Figure 5A), demonstrated early activation of SA-biosynthesis in *cpr1-4*. The concentrations of the wounding hormone jasmonate and its isoleucine-conjugate did not differ significantly between T19-2 and *cpr1-4* (Figure 5A). The levels of abscisic acid (ABA), which is a repressor of *2CPA* expression and of many other nuclear genes for chloroplast proteins and plastid genes (Baier et al., 2004; Staneloni et al., 2008; Yamburenko et al., 2013), were also similar at 14 and 28 d, and decreased in *cpr1-4* at 42 d (as compared to T19-2), excluding any relevance of ABA with respect to low *2CPA* activation in *cpr1* in early stages.

### Developmental regulation of the *PAS* and *PR* genes in the *cpr1-4* mutant

To analyse how CPR1 affects defense and plastid antioxidant signaling, we compared the expression of *2CPA, MDAR* and *tAPX* (PAS genes) with *PAD4, SID2*, and *PR2* (defense genes) in leaves of 14-, 28-, and 42-day-old *cpr1-4* and T19-2 by qRT-PCR (Figure 5B; data for PAS-gene regulation in *cpr1-5*: Supplementary Figure 3). In *cpr1-4*, the transcript levels of the three PAS genes were slightly reduced in 14- and 28-day-old plants, but were similar to T19-2 at 42 days. The expression of the three defense genes was higher in *cpr1-4* mutants than in T19-2 at all developmental time points (Figure 5B). For *PAD4* and *SID2*, the difference was strongest in 28-day-old plants.

### *cpr1-4* accumulates more ROS and activates ROS signaling stronger than wildtype plants

**Figure 6:**
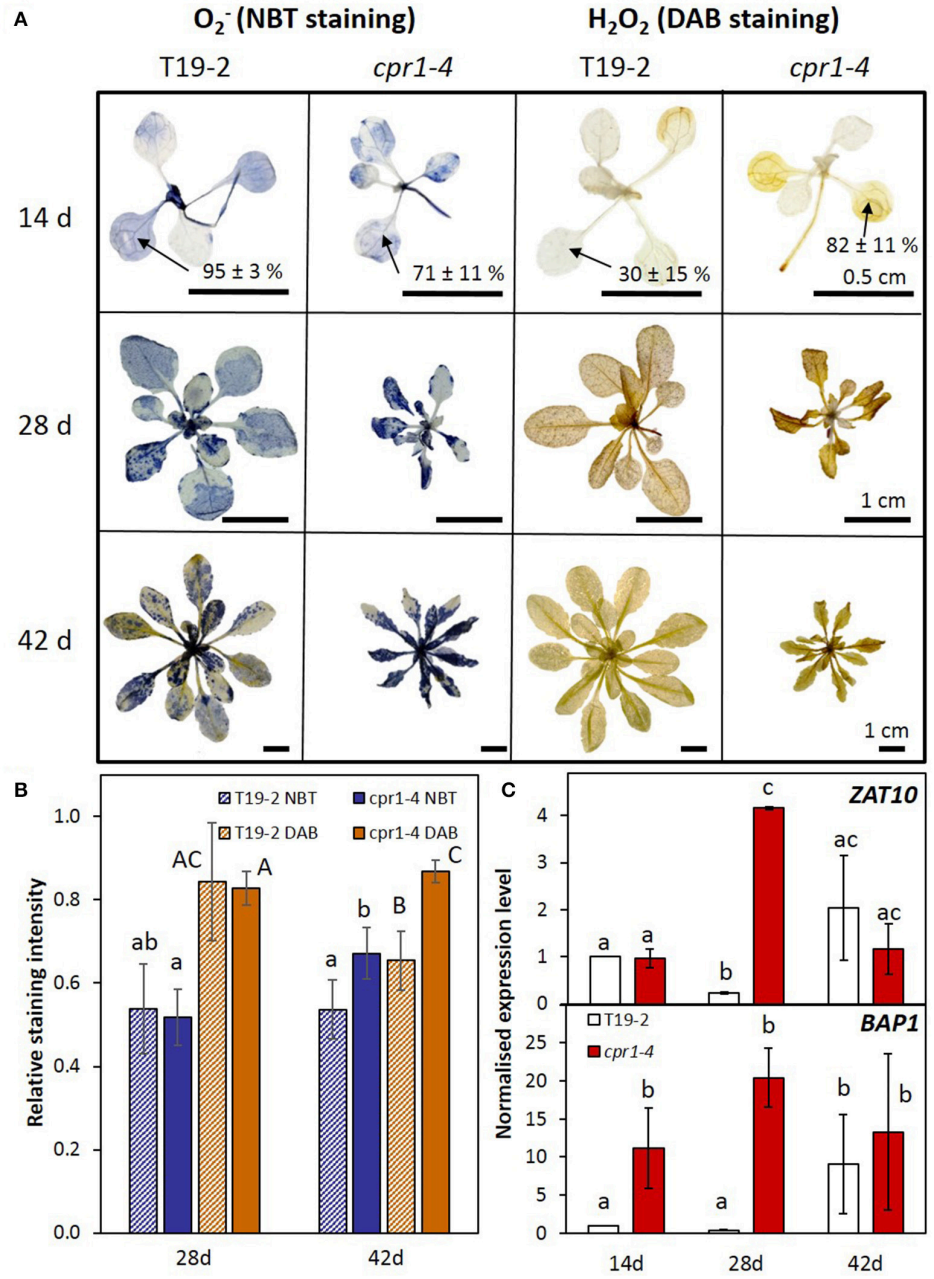
ROS metabolism and signaling in *cpr1* mutant and T19-2 plants during development. **(A)** Detection of ${\text{O}}_{2}^{-}$ and H_2_O_2_. Representative pictures from two experiments with five replicates each. For 2-week-old plants, the staining intensity in cotyledons was determined. The percentages (means ± s.d.) show staining intensities in the cotyledons. **(B)** ${\text{O}}_{2}^{-}$ and H_2_O_2_ content per rosette area as determined by NBT- and DAB-staining (ANOVA, *P* < 0.05). **(C)** Normalized transcript levels of ROS marker genes *BAP1* and *ZAT10* in *cpr1-4* and T19-2 plants during development. Bars represent the mean and standard errors of three biological replicates, each measured in triplicate and normalized to *At5g15710* (*F-box*) and *At5g08290* (*YLS8*) transcript levels (Statistically significant differences are labeled with different letters; ANOVA, *P* < 0.1).

To study the consequences of the *cpr1-4* mutation and insufficient antioxidant protection on ROS-metabolism, we analyzed the levels of the two major ROS, ${\text{O}}_{2}^{-}$ and H_2_O_2_ (Figure 6). Generation of ${\text{O}}_{2}^{-}$ at the thylakoids (Mehler, 1951) increases upon excess excitation pressure (Foyer et al., 1994). c*pr1-4* showed higher ${\text{O}}_{2}^{-}$ and H_2_O_2_ levels in the cotyledons of 14-day-old plants than T19-2 (Figure 6A). At an age of 28 d, some staining patterns indicated higher ROS-levels in *cpr1-4* (Figure 6A). Quantification of a series of plants showed high variability and indistinguishable mean values between T19-2 and *cpr1-4* (Figure 6B). Later, at 42 d, the ${\text{O}}_{2}^{-}$ and H_2_O_2_ levels again increased to significantly higher ones than in T19-2 (Figure 6B).

*ZAT10*, which responds to various types of ROS of plastidic and extra-plastidic origin, such as to ozone, H_2_O_2_ and O_2_^−^ (Rossel et al., 2007), was strongly induced in 28-day-old *cpr1-4* plants (Figure 6C) and in *cpr1-5* plants of all age (Supplementary Figure 3). The ROS-inducible gene *BAP1* responds to transfer of excess energy from pigments to oxygen at the thylakoid membrane (op den Camp et al., 2003). It was expressed more strongly in leaves of 14- and 28-day-old *cpr1-4* plants than those of T19-2 (Figure 6C) and highly accumulated in 14, 28, and 42 day old *cpr1-5* as compared to the respective wildtype (Supplementary Figure 3).

### *Specificity and causality of cpr1* on the regulation of genes for chloroplast proteins

**Figure 7:**
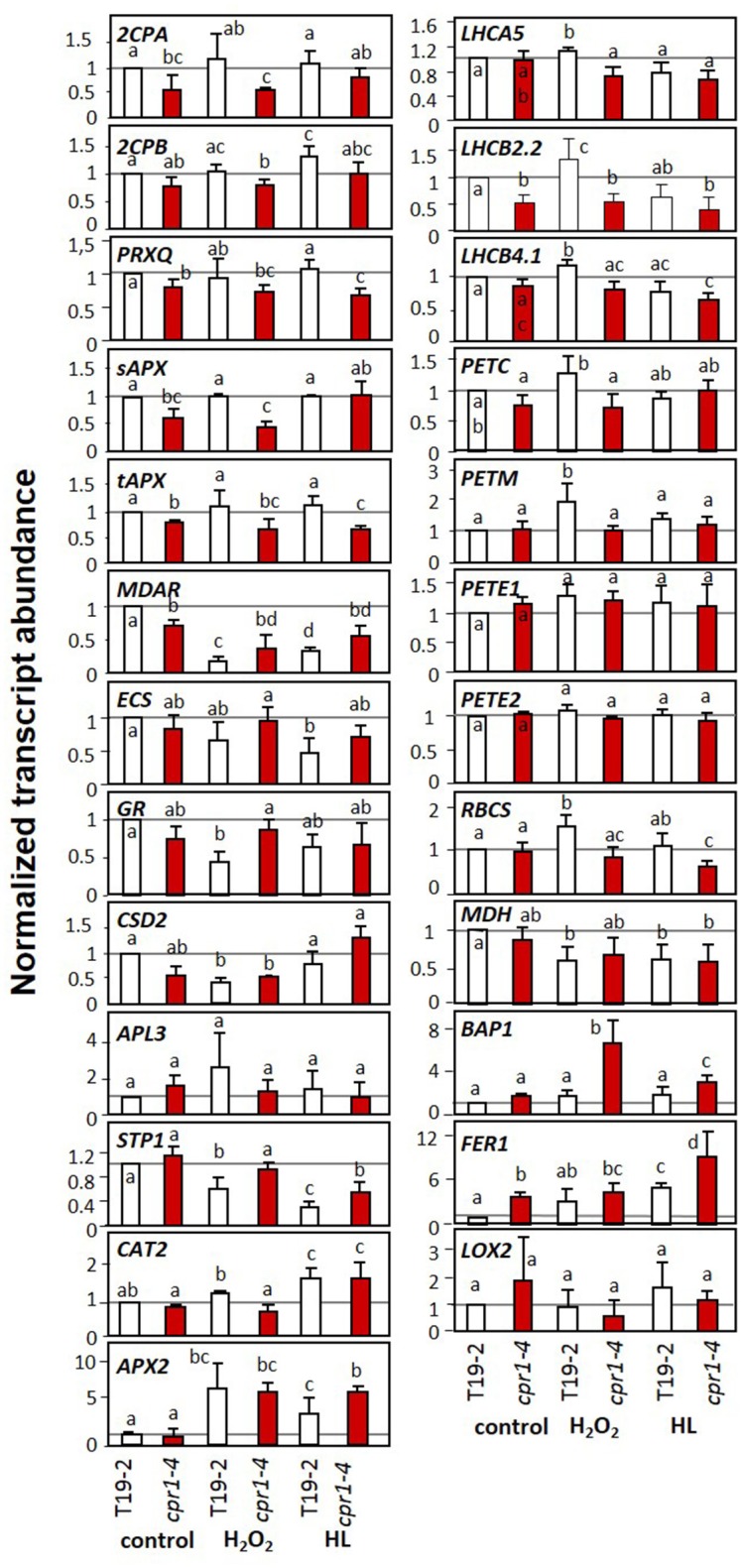
Transcript levels of genes encoding chloroplast antioxidant enzymes, photosynthetic proteins, sugar-related enzymes and marker genes for oxidative stress and wounding upon H_2_O_2_ and high light (HL) treatment in 9-day-old seedlings of *cpr1-4* and T19-2 grown in sterile culture. The seedlings were infiltrated with 10 mM H_2_O_2_ or exposed to 800 μmol photons m^−2^ s^−1^ (HL) while floating on MS-medium for 4 h. Bars represent the mean and standard deviation of four to seven biological replicates by qRT-PCR and normalized to *actin2* (*At3g18780)* transcript levels. Statistically significant differences are indicated with different letters (ANOVA, *P* < 0.1).

The *cpr1-4* (*rimb6*) mutant was isolated for low activation of *2CPA* promoter activity at the seedling stage and shown to be affected in expression of other PAS genes in 3 week old soil grown plants (Heiber et al., 2007). To test the target spectrum and the impact of elevated ROS levels, we investigated the regulation of a series of *PAS* genes, genes for light-harvesting complex proteins (*LHCA* and *LHCB*), for photosynthetic electron transport components (*PET* genes), ribulose-1,5-bisphosphate carboxylase small subunit (*RBCS*), sugar metabolism proteins (*APL3* and *STP1*), stress marker genes (*BAP1* and *FER1*) and extra-plastidic antioxidant enzymes (*APX2* and *CAT2*) in T19-2 and *cpr1-4* in response to externally applied H_2_O_2_ and to high light (HL) in 9-day-old seedlings on MS-medium optimized for seedling growth and minimal impact on greening and PAS gene expression (Heiber et al., 2014) (Figure 7). The *cpr1-4* mutant showed significantly lower expression of genes for chloroplast peroxidases, monodehydroascorbate reductase (*MDAR)* and *LHCB2.2* under control conditions. The transcript levels of the ROS-regulated genes, *BAP1* (Bachmann et al., 2002; op den Camp et al., 2003; Spoel et al., 2003) and *Fer1* (Bachmann et al., 2002; op den Camp et al., 2003; Spoel et al., 2003), were slightly (*BAP1*) or significantly (*Fer1*) increased in *cpr1-4* mutants under control conditions (Figure 7).

*CAT2* (catalase) and *APX2* (ascorbate peroxidase), which encode ROS-inducible extra-plastidic antioxidant enzymes (Mullineaux et al., 2000; Du et al., 2008), responded to the HL and H_2_O_2_ treatment in T19-2, but showed no difference between T19-2 and *cpr1-*4 under control conditions.

In *cpr1-4*, the transcript levels of *PrxQ, tAPX*, and *LHCB2.2* were lower in control plants and in H_2_O_2_- and HL-treated plants (Figure 7). For PrxQ and tAPx, the mRNA levels were slightly decreased in H_2_O_2_-treated plants and significantly lower in response to HL. The transcript levels of the two peroxiredoxin genes *2CPA and 2CPB*, of *sAPx* and of CuZn superoxide dismutase 2 (*CSD2*) were lower in H_2_O_2_-treated *cpr1-4* than in *cpr1-4* under control conditions. Moderate HL resulted in higher transcript levels. Antagonistic regulation by light and H_2_O_2_ is consistent with the regulatory model postulated for *2CPA* based on identification of distinct promoter motifs (Baier et al., 2004) and analysis of redox-box regulation by the transcription factor RAP2.4a (Shaikhali et al., 2008).

**Figure 8:**
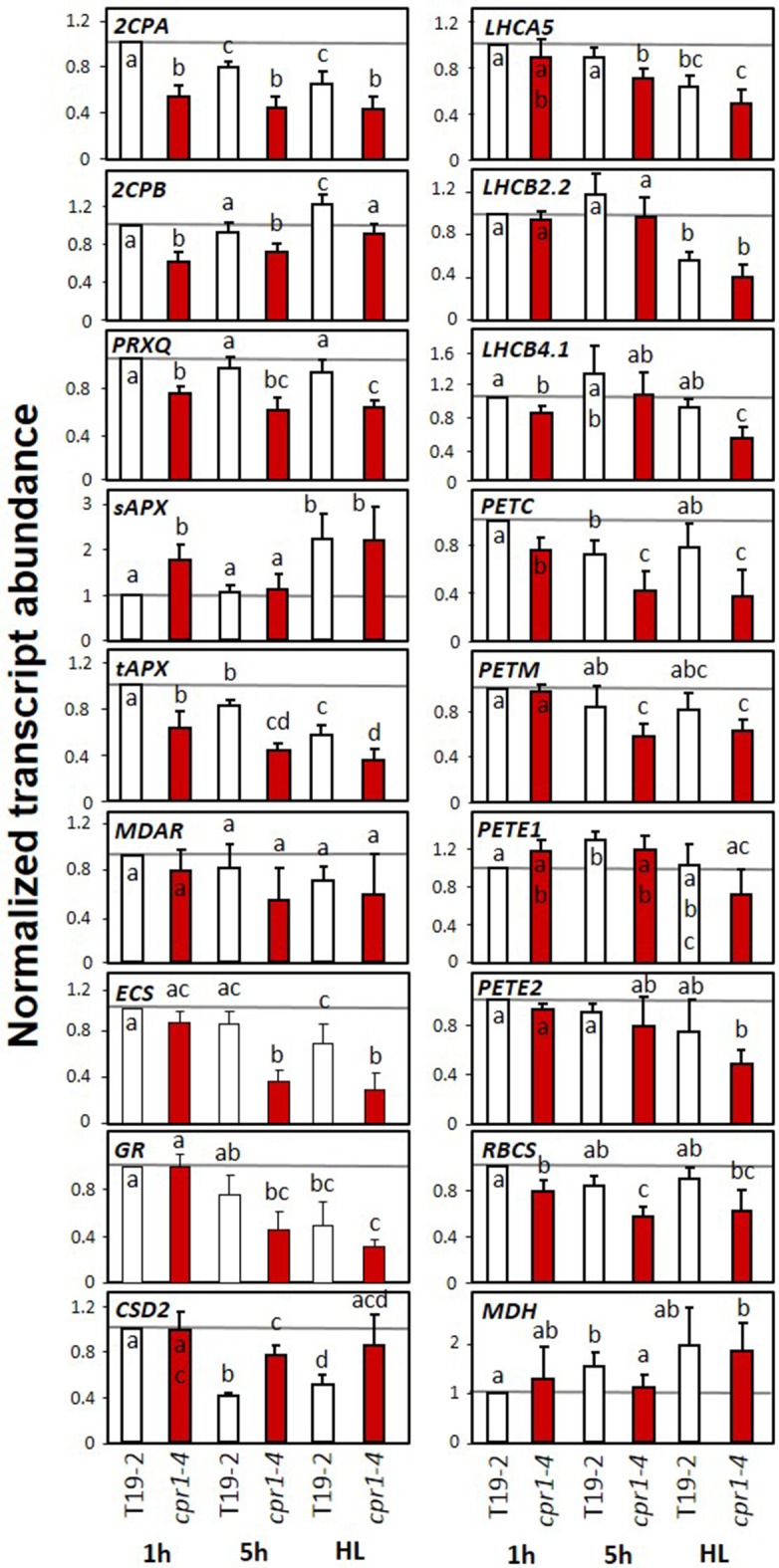
Transcript levels of genes encoding chloroplast antioxidant enzymes, photosynthetic proteins, sugar related enzymes and marker genes for oxidative stress and wounding in 4-week old, sterile grown *cpr1-4* and T19-2 in response to high light (HL). Four-week-old plants were harvested 1 and 5 h after exposure to 100 μmol photons m^−2^ s^−1^ or after exposure to 800 μmol photons m^−2^ s^−1^ (HL) for 4 h starting 1 h after the beginning of the light-phase. Bars represent the mean and standard deviation of four to seven biological replicates by qRT-PCR and normalized to actin2 (*At3g18780)* transcript levels. Statistically significant differences are labeled with different letters (ANOVA, *P* < 0.1).

In 28-day-old sterile grown plants, the transcript levels of most PAS, PET and LHC genes and of *RBCS* were at least slightly more decreased after 4 h HL treatment than in the control plants harvested 1 h after onset of light (Figure 8). However, for *tAPX, ECS, GR, PETC* and *PETM* the HL effect was not distinguishable from the effect of additional 4 h at normal light intensity. *sAPX* was expressed at higher levels in *cpr1-4* than in T19-2 1 h after onset of light and indistinguishable from T19-2 after 5 h in standard growth light or HL. Although *sAPX* and *tAPX* are coregulated by RAP2.4 transcription factors (Rudnik et al., 2017), *sAPX* is often inversely regulated to *tAPX* in response to stress or metabolite availability (Heiber et al., 2014; Juszczak et al., 2016). *CSD2* transcript levels were not decreased 1h after onset of light and were less decreased after 5 h illumination in *cpr1-4* than in T19-2.

### Ascorbate and glutathione status in *cpr1-4* under H_2_O_2_ and light treatments

**Figure 9:**
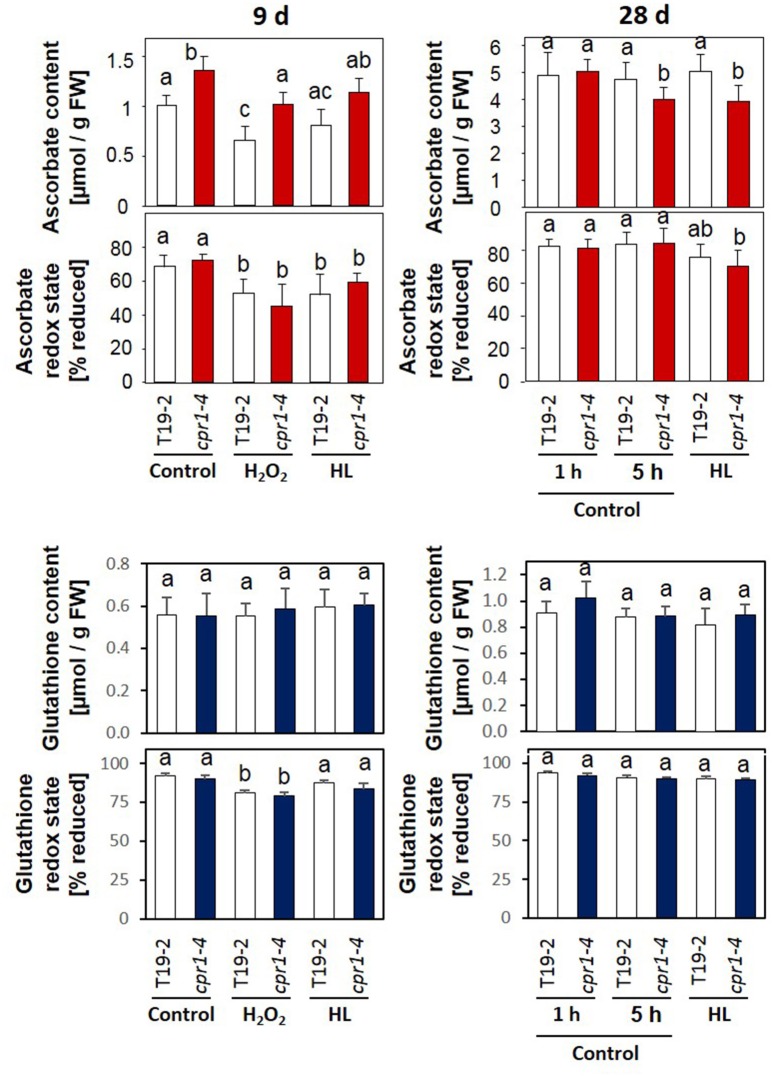
Ascorbate and glutathione contents and redox states in 9-day-old seedlings and 4-week-old rosette plants. Sterile grown 9-day-old seedlings were infiltrated with 10 mM H_2_O_2_ or exposed to 800 μmol photons m^−2^ s^−1^ (HL), while floating on MS-medium for 4 h. The 4-week-old plants were exposed to 100 μmol photons m^−2^ s^−1^ (control conditions) or 800 μmol photons m^−2^ s^−1^ (HL) for 4 h starting 1 h after onset of the light-phase to which the plants were acclimated. The ascorbate and glutathione contents and the redox states (amount of reduced form/total amount of glutathione or ascorbate) of the ascorbate pool were determined for 8–12 plants per treatment and genotype. Statistically significant differences are labeled with different letters (ANOVA, *P* < 0.1). Dehydroascorbate/ascorbate and GSSG/2× GSH ratios calculated from the same data are depicted in Supplementary Figure 4.

Ascorbate biosynthesis depends on carbohydrate availability and stress activation (Bartoli et al., 2003; Pena-Ahumada et al., 2006; Heiber et al., 2014). The ascorbate levels were higher in 9-day-old *cpr1-4* plants than in T19-2 (Figure 9). Four hours of incubation with H_2_O_2_ or illumination with 800 μmol photons m^−2^ s^−1^ (HL) had similar effects on ascorbate consumption and ascorbate oxidation in both genotypes (Figure 9 and Supplementary Figure 4). During further rosette development, ascorbate accumulated. In 28-day-old *cpr1-4*, the reduction state of the ascorbate pool was attenuated (Figure 9). However, the ascorbate pool size was decreased in *cpr1-4* after 5 h illumination with growth light intensity (NL) and HL (Figure 9) demonstrating increased consumption.

Transcript abundance analysis showed stronger expression of *GR* and slightly stronger expression of *ECS* (γ-glutamyl-cysteine synthase), which are involved in glutathione reduction and biosynthesis, respectively, in 9 day old *cpr1-4* mutants in response to H_2_O_2_ application and lower expression in 28 day old *cpr1-4* after 5 h illumination with standard light and high intensities demonstrating that the regulatory effect is light intensity independent. Quantification of the glutathione content and determination of the redox state of the glutathione pool (Figure 9) and calculation of the GSSG/GSH ratio (Supplementary Figure 4) demonstrated that the glutathione pool was, in contrast to the ascorbate pool, not significantly affected in *cpr1-4*.

### Impact of regulators of pathogen defense response on the expression of chloroplast antioxidant enzymes

Various PAS genes and pathogen response genes were regulated inversely in *cpr1-4* as compared to T19-2 (Figure 6). To test whether the expression of the immune regulators affect PAS gene expression in absence of pathogens, as CPR1 does, we analyzed the transcript levels of *2CPA, MDAR*, and *sAPX* in comparison to *CPR1* and the defense genes *PAD4* and *PR2* during development in *wt* plants and the immune signaling mutants *npr1* (Cao et al., 1997), *pad4* (Jirage et al., 1999), and *rps2* (Cheng et al., 2011) at three developmental stages.

**Figure 10:**
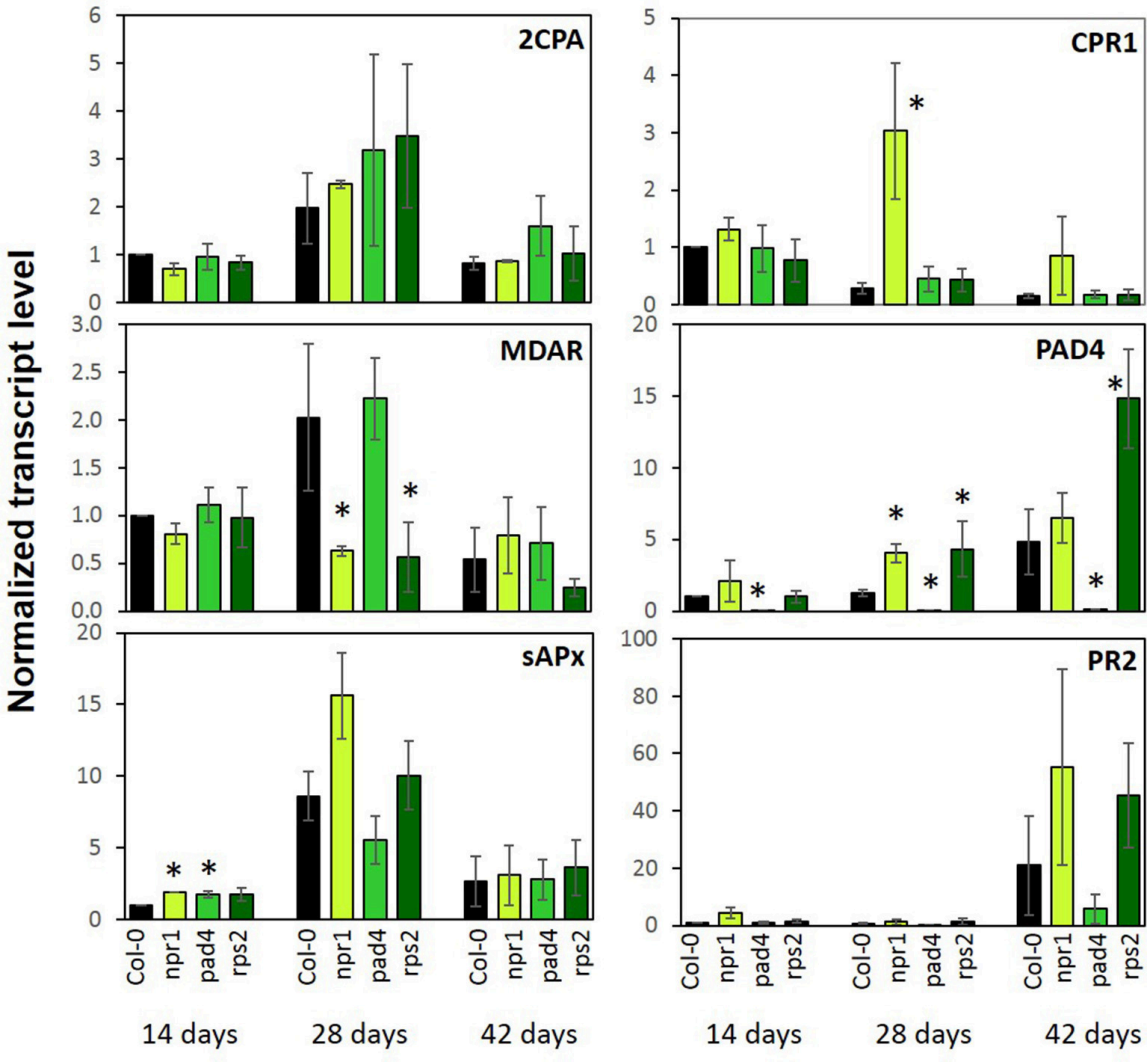
Transcript levels of genes encoding chloroplast antioxidant enzymes and marker genes for the pathogen defense response in 2-, 4-, and 6-week old *npr1, pad4, rps2*, and *wildtype* (Col-0). Bars represent the mean and standard error of three biological experiments, each measured in triplicate by qRT-PCR and normalized to *At3g18780* (F-box) and *At5g15710* (YLS8). Statistically significant differences between developmental time points for a single genotype are indicated with an asterisk (ANOVA, *P* < 0.1).

*CPR1* transcript levels decreased with age in Col-0 (Figure 10). *PAD4* levels tended to increase. The wildtype *CPR1* pattern was maintained in the *pad4* and *rps2* mutants. In the *npr1* mutant, *CPR1* transcript levels were increased as compared to Col-0 at an age of 28 d and 42 d. The *PAD4* mRNA levels were elevated in *npr1* at 28 d and in *rps2* at 28 d and 42 d.

As a read-out for induction of immunity responses, PR2 transcript levels were analyzed. They increased in all genotypes at an age of 42 d, but reached higher levels in *npr1* and *rps2* and lower levels in *pad4* compared to Col-0.

The PAS genes *2CPA, sAPX* and *MDAR* showed highest expression at 28 days in Col-0. *2CPA* expression, which was the reporter used for selecting the *rimb*-mutants (Heiber et al., 2007), was not significantly affected in any of the three immune mutants (Figure 10). *sAPX* showed slightly higher levels in *npr1* and *pad4* mutants in 14-day-old seedlings, but the general developmental pattern was maintained. *MDAR* expression showed the *wt* developmental pattern in *pad4*, but not in *npr1* and *rps2* (Figure 10). None of the genes for PAS enzymes was disregulated in these defense signaling mutants to the same extent as in *cpr1-4* (Figures 5, 10). We conclude that the CPR1-mediated regulation of defense responses and PAS genes occur independently and this partially depends on the developmental stage of the plants.

**Figure 11:**
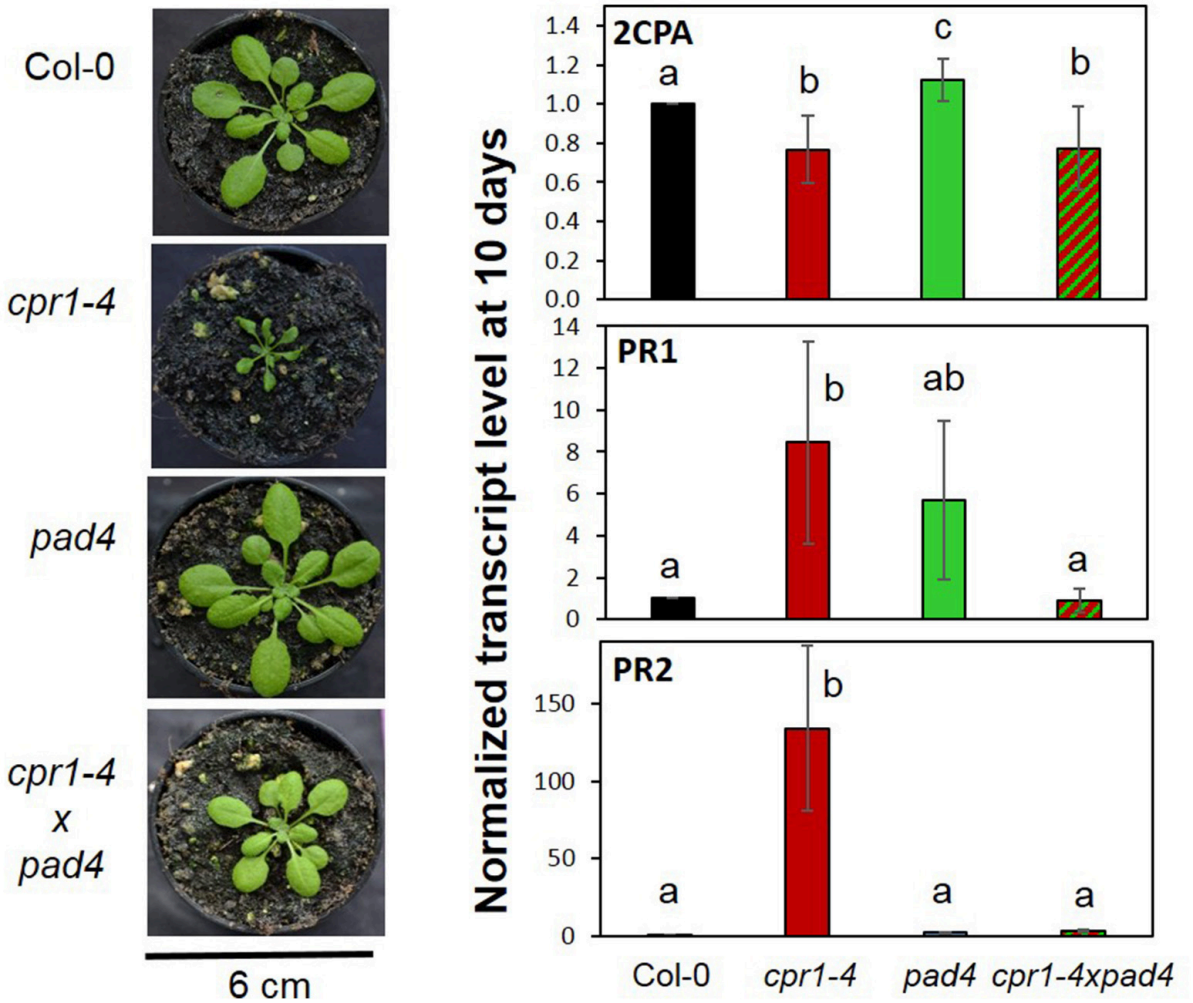
Effect of PAD4-mediated SA induction on the habitus and gene expression regulation of *cpr1-4. cpr1-4* was crossed with *pad4. cpr1-4xpad4* double mutants were selected from the F_2_ population. **(Left)** Habitus of 28-day-old *cpr1-4, pad4*, and *cpr1-4xpad4* mutants compared to Col-0 wildtype plants. **(Right)** *2CPA, PR1*, and *PR2* transcript levels in 10-day-old Col-0, *cpr1-4, pad4*, and *cpr1-4xpad4* plants grown aseptically on MS-medium. The means were calculated from 3 technical replicates per probe and 1–3 probes per 3 independently grown plant sets. For each probe, RNA was extracted from 50 seedlings. Statistically different results are indicated for each gene with different letters (ANOVA, *p* < 0.01).

To test the hypothesis, we compared *2CPA, PR1*, and *PR2* expression in wildtype plants, *cpr1-4* and *pad4* single and *cpr1-4* x *pad4* double mutants after 10 days of growth on 0.5 MS medium, which were the conditions for the *rimb*-mutant screen (Heiber et al., 2007). *pad4* was crossed into the *cpr1-4* background to avoid activation of the SA biosynthesis, while maintaining basic SA biosynthesis and SA sensitivity (Zhou et al., 1998). The *pad4* mutation widely restored the leaf habitus and rosette growth defects of *cpr1-4* (Figure 11). At an age of 28 days, the rosette diameter of *cpr1-4* mutants was about 50% of wildtype Col-0 and had 29.5 ± 3.7% fewer leaves, while the double mutants were only 14.3 ± 2.4% smaller in diameter and had formed on average 2.1 ± 0.5 fewer leaves than Col-0.

Despite the high data noise due to low expression intensity of *PR1* and *PR2* in wildtype plants, the transcript levels of *PR1* and *PR2* were significantly (pairwise *t*-test; *p* < 0.01) increased in the *cpr1-4* mutants. The strong induction effect was lost in *cpr1-4* x *pad4* double mutants, demonstrating that transcript accumulation of both genes depends on activation of the PAD4-mediated SA loop (Figure 11). On the contrary, lack of PAD4 did not (significantly) affect the *cpr1-4* mutant effect on *2CPA* expression (Figure 11), showing that low expression of *2CPA* in the *cpr1-4* mutant is independent from SA accumulation.

## Discussion

CPR1 is part of an SCF-E3-ubiquitin ligase complex. As an F-box protein, it controls the substrate specificity. It was shown experimentally to mediate the proteasomal turnover of the R-proteins SNC1 and RPS2 (Gou et al., 2009, 2012; Cheng et al., 2011) and other proteins (Wang et al., 2014). R-protein degradation counteracts induction of effector triggered immunity (Clarke et al., 2001; Jirage et al., 2001). Furthermore, CPR1 regulates microtubule arrangement (Han et al., 2015) and supports degradation of the chloroplast Hsp100 chaperon ClpC1 and of glutamine synthase 1 (Wang et al., 2014). Mapping of the *rimb6* mutation to the *CPR1* locus identified *rimb6* as a *cpr1* allele (Figure 2) and revealed that CPR1 is also essential for full transcriptional activation of PAS genes and genes encoding components of photosynthetic electron transport chain and for carbon assimilation in absence of pathogens (Figures 7, 8). This function is linked to avoiding and antagonizing ROS accumulation (Baier and Dietz, 1999b; Baier et al., 2000; Heiber et al., 2007; Kangasjärvi et al., 2008; Pulido et al., 2009). The effects of CPR1 on PAS gene expression were strongest in young plants and preceded the maximum effect on the SA-biosynthesis-related genes *PAD4* and *SID2* (Zhou et al., 1998; Wildermuth et al., 2001) (Figure 5B).

Although expression of various PAS genes was decreased, the antioxidant system was not massively overwhelmed in 14 and 28 day old plants (Figure 6). Increased ROS levels could only be detected in the oldest leaves by ROS staining, which integrates information on ROS-levels over time (Figure 6). However, the highly ROS-sensitive marker gene *BAP1* (op den Camp et al., 2003; van Buer et al., 2016), which responds to increased chloroplast ROS production by the EXECUTER-regulated chloroplast-to-nucleus signaling pathway (Lee et al., 2007), was increased in 14, 28, and 42 day old *cpr1-4* (Figure 6B). The other chloroplast ROS marker gene *ZAT10* (Mittler et al., 2006; van Buer et al., 2016) was increased from 28 days onwards in *cpr1-4* and even earlier and stronger in the T-DNA insertion line *cpr1-5* (Supplementary Figure 3). Insufficient antioxidant protection in *cpr1* mutants coincided also with higher reduction states of metabolites (Figure 4), and early accumulation of the low molecular weight antioxidant ascorbate (Figure 9). Cellular redox and metabolite imbalances lead to microtubule disaggregation (Livanos et al., 2012) and could explain the cell shape defects, as observed by Han et al. (2015) and in this study (Figure 1E), as a redox imbalance effect in absence of pathogens. The main question arising from the identification of *rimb6* as a mutant allele of CPR1 is how the signal transduction pathways suppressing immune defense and activating chloroplast antioxidant protection are linked.

Since activation of ETI decreases chloroplast function (Zimmerli et al., 2004; Prokopova et al., 2010; Kyselakova et al., 2011), it is tempting to assume that the CPR1-controlled immune signaling pathway directly or indirectly controls PAS gene expression. To test this hypothesis, we analyzed PAS gene expression in a selection of loss of function mutants of ETI-mediating factors, including the *rps2* mutant, which is defective for the RPS2 protein that is normally negatively regulated by CPR1 (Cheng et al., 2011), and in *npr1* and *pad4* mutants which are impaired in downstream components of ETI signaling (Cao et al., 1997; Jirage et al., 1999; Zhang et al., 2003). In our study, the experiments were performed in absence of pathogens to avoid CPR1-independent pathogen-induced ETI effects. PAS gene expression was not significantly affected in *rps2* mutants (Figure 10) excluding that the CPR1 target RPS2 promotes PAS gene expression in absence of pathogens. If the expression of PAS genes are inversely regulated by NPR1 or PAD4, then a lack of these factors should increase their transcript levels. However, no effect (as compared to *wt*) was observed for *2CPA*, which was the reference gene for isolation of the *cpr1-4* (*rimb6*) mutant in the *rimb*-screen (Heiber et al., 2007), in *npr1* and *pad4* mutants on soil (Figure 10). Only in 10-day-old seedlings under aseptic conditions, slightly higher 2CPA transcript levels were observed in the *pad4* mutant (Figure 11). sAPx transcript levels were increased only in 14-day-old plants and MDAR transcripts were even decreased in 28-day-old plants (Figure 10).

ETI and its CPR1-dependent regulation are widely associated with SA accumulation (White, 1979; Lovelock et al., 2016; Van Wersch et al., 2016). SA mediates local and systemic protection against biotic and abiotic stress (Durrant and Dong, 2004). Furthermore, accumulation of SA causes dwarfism (Van Wersch et al., 2016) and affects thylakoid organization, the redox state of the plastoquinone pool (Gawronski et al., 2013), ROS-signaling (Rivas-San and Plasencia, 2011) and the activity of catalase and chloroplast and cytosolic ascorbate peroxidases (Durner and Klessig, 1995). Consequently, the low *2CPA* expression in *cpr1/rimb6* mutants could be due to indirect secondary effects of SA accumulation on the cellular redox poise and redox signaling (Figures 4B, 7, 8), although the *2CPA* promoter is insensitive to short-term SA treatments (Heiber et al., 2007). The importance of SA and its effect on plant development (Van Wersch et al., 2016) and *2CPA* expression were tested by crossing the *pad4* mutation into the *cpr1-4* background (Figure 11). PAD4 functions upstream of SA biosynthesis (Zhou et al., 1998; Feys et al., 2001). While basal SA biosynthesis and SA sensing are unaffected, the *pad4* mutant does not accumulate SA (Zhou et al., 1998; Feys et al., 2001). The high expression levels of *PR1* and *PR2* (observed in *cpr1-4* mutants; Figure 5B) were reduced to wildtype levels in the *cpr1-4 x pad4* double mutant (Figure 11) demonstrating the effect of SA on regulation of the ETI genes. On the contrary, the *2CPA* transcript level in the *cpr1-4* x *pad4* double mutant was similar to the *cpr1-4* single mutant (Figure 11). We conclude that the *cpr1-4* effect on *2CPA* expression is independent of PAD4-mediated immune signaling and therefore the regulation of *2CPA* is independent from SA biosynthesis.

*CPR1* expression is strongest in young seedlings (Figure 10), which are exceptionally sensitive to redox and metabolite imbalances (Sanchez-Fernandez et al., 1997; Francis and Halford, 2006; Hiltscher et al., 2014; Schippers et al., 2016) because antioxidant protection is limiting (Pena-Ahumada et al., 2006). ROS-triggered activation of SA biosynthesis increases the risks of damage (Kangasjärvi et al., 2005; Gou et al., 2009) (Figure 5A). The support of PAS activation by CPR1 in wildtype plants (Figures 3B, 5, 7, 8, 10) can help to protect the young tissues from accumulation of ROS (Figure 6), and the subsequent inappropriate activation of ROS-signaling (Figure 6) and defense responses (Figure 11). ROS accumulation in *cpr1* mutants (Figure 6) disturbs cell development (Han et al., 2015; Figure 1) and contributes to protein oxidation (Heiber et al., 2007). ROS damage of PAS enzymes (Heiber et al., 2007; Baier et al., 2010), further increases ROS accumulation (Figure 6) until ROS levels reach a threshold that will activate defense signaling (Figures 5B, 6B). As a side-effect of these processes, the expression of a wider set of genes for plastid proteins becomes disregulated (Figures 7, 8), and H_2_O_2_ accumulates throughout the rosettes in older *cpr1-4* mutants (Figure 6B—42 day old plants). In parallel, the expression of two main drivers of SA biosynthesis, *SID2* and *PAD4* (Zhou et al., 1998; Wildermuth et al., 2001), increases (Figures 5, 10). Accumulation of SA further promotes defense activation.

From comparison of *PR1, PR2* and *2CPA* regulation in *cpr1-4* single and *cpr1-4* x *pad4* double mutants (Figure 11) we conclude that PAS and ETI are regulated by differentially controlled CPR1-dependent signaling cascades. Very little is known about CPR1 functions apart from its effect on ETI (Jirage et al., 2001; Cheng et al., 2011) and cell shape (Han et al., 2015). Proteome comparisons identified the plastid-localized Hsp100 chaperone ClpC1 as one of two proteins that are directly destabilized by CPR1 (Wang et al., 2014). The other was glutamine synthase 1 (GSR1), which is involved in plastid N-assimilation and might explain the higher amination status in *cpr1-4* mutants (Figure 4). ClpC1 is an anti-chlorosis factor and stabilizer of photosynthesis proteins (Sjorgen et al., 2004). Higher availability of ClpC1 may explain why *cpr1-4* leaves stay green for longer than *rimb1/rcd1-6* leaves (Heiber et al., 2007), but does not explain the lower expression of PAS genes relative to *RBCS* and *LHC*- and *PET*-genes, which are under control of the turn-over of chlorophyll a/b-binding proteins via tetrapyrrole-signaling (Strand et al., 2003; Nott et al., 2006; Figures 7, 8).

**Figure 12:**
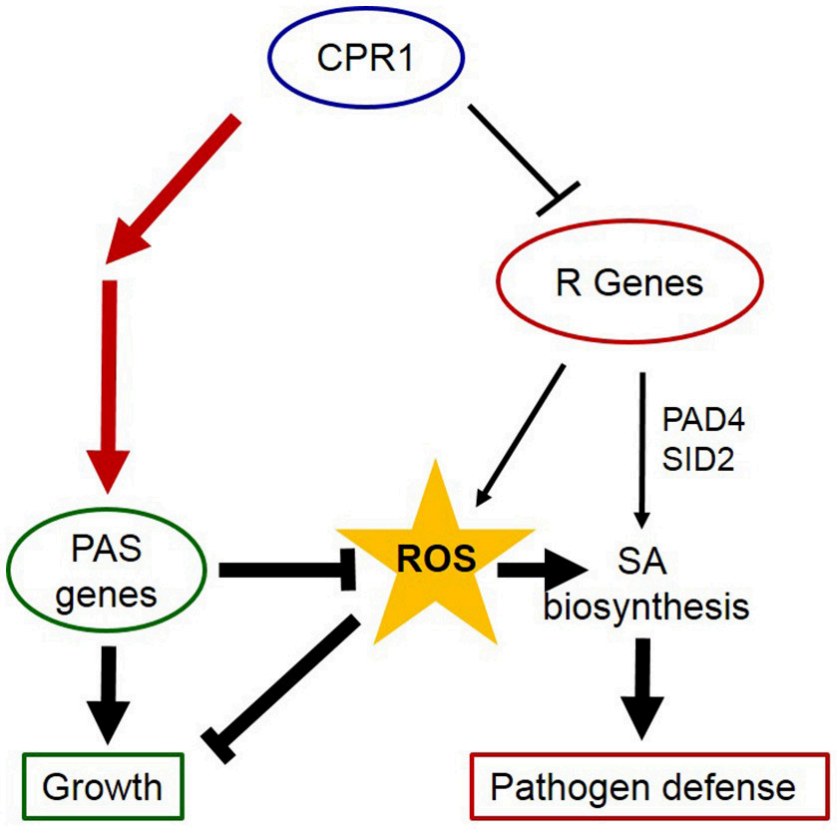
CPR1 supports growth and avoids activation of pathogen responses via regulation of PAS gens. CPR1 antagonizes activation of ETI in absence of pathogens by marking R-genes for degradation (thin lines). Via their impact on the cellular ROS levels, PAS enzymes impact on SA-biosynthesis and induction of ETI (bold lines). Here, we showed that CPR1 is essential for full induction of PAS genes and that CPR1-controlled PAS regulation is independent of SA accumulation (red lines). We conclude that CPR1 controls two interacting signaling pathways, in which full expression of PAS genes antagonizes accumulation of ROS, which are signals activating biotic and abiotic stress responses. The chloroplast loop is supposed to help wildtype plants to avoid activation of cost-intensive and growth limiting defense reactions in absence of biotic and abiotic stressors.

The PAS is an active system antagonizing ROS accumulation and maintaining the cellular redox homeostasis (Asada, 2006). Most PAS genes, first of all *2CPA*, are highly expressed even under non-stress conditions (Baier and Dietz, 1997; König et al., 2002; Baier et al., 2010). Slight redox imbalances, as observed for ascorbate and glutathione (Figure 9) keep the activating transcription factor Rap2.4a in its active dimeric form and PAS gene expression high (Shaikhali et al., 2008). Our study demonstrated that the cytosolic CPR1 (Gou et al., 2009; Cheng et al., 2011) is necessary for the full induction of PAS genes and genes for other chloroplast proteins. The effect of CPR1 on PAS may indirectly affect ETI responses. In nature, ETI occurs as a result of receptor-mediated pathogen recognition (Jones and Dangl, 2006) and this activates ROS synthesis (Zimmerli et al., 2004; Prokopova et al., 2010; Kyselakova et al., 2011) and stabilizes pathogen response reactions (Sharma et al., 1996). Various experiments have demonstrated, that ROS signals, e.g., due to photooxidation or lower detoxification of chloroplast ROS, can stimulate ETI responses in absence of pathogens (Mühlenbrock et al., 2008; Straus et al., 2010; Han et al., 2013a,b). Consistently, we hypothesize that the effect of CPR1 on the control of chloroplast function (Figures 7, 8) and stabilization of the cellular redox and metabolite homeostasis (Figures 4, 9) prevents inappropriate activation of defense responses in the absence of pathogens and supports CPR1-controlled suppression of R-protein mediated induction of ETI (Figure 12).

## Conclusion

CPR1 is involved in full activation of PAS gene expression in young leaves. The SA-insensitive chloroplast antioxidant protection system antagonizes ROS accumulation and subsequent stimulation of SA-biosynthesis, which otherwise could activate ETI (Figure 12). We conclude that CPR1-dependent regulation of *2CPA* (and other PAS genes) serves as a reinforcement mechanism supporting R-protein mediated suppression of ETI in absence of pathogens.

## Accession numbers

*2CPA* (At3g11630), *2CPB* (At5g06290), *Actin2* (At3g18780), *APL3* (At4g39210), *APX2* (At3g09640), *BAP1* (At3g61190), *CAT2* (At4g35090), *CSD2* (At2g28190), *ECS* (At4g23100), *F-Box* (At5g15710), *FER1* (At5g01600), *GR* (At3g54660), *LHCA5* (At1g45474), *LHCB2.2* (At2g05070), *LHCB4.1* (At5g01530), *LOX2* (At3g45140), *MDAR* (At1g63940), *MDH* (At5g58330), *NPR1* (At1g64280), *PAD4* (At3g52430), *PETC* (At4g03280), *PETE1* (At1g76100), *PETE2* (At1g20340), *PETM* (At2g26500), *PR1* (At2g14610), *PR2* (At3g57260), *PrxQ* (At3g26060), *RBCS* (At5g38430), *RIMB6/CPR1* (At4g12560), *RPS2* (At4g26090), *sAPX* (At4g08390), *STP1* (At1g11260), *SID2* (At3g62030), *SNC1* (At4g16890), *tAPX* (At1g77490), *YLS8* (At5g08290), *ZAT10* (At1g27730)

## Author contributions

CH and ER genotyped *cpr1-4* and *cpr1-5*, did the qRT-PCRs depicted in Figures 2, 5, 6, 11. CH performed also the ROS staining experiments and drafted parts of the manuscript and figures. ER also did the habitus documentation, crossed the mutants and selected the lines for further analysis, performed the CAPS marker analysis and the qRT-PCRs depicted in Figures 3, 10. HH started SSLP-mapping and performed the electron microscopy. WG finalized the SSLP-mapping and performed high-throughput sequencing and mapping analysis with BR. IH performed the ascorbate measurements, the qRT-PCRs depicted in Figures 7, 8, prepared the samples for the GC-MS analysis and drafted the figures. MB supervised the glutathione measurements on plant material prepared by IH in a lab training course for students of the Carl-Severing-Berufskolleg (Bielefeld), did the calculations and prepared the figures. AB and KN did the GC-MS-analysis. TL and AS performed the phytohormone quantification and gave advice on the statistical analysis. JvB quantified the ROS levels. MB supervised the project and finalized with BR the manuscript.

### Conflict of interest statement

The authors declare that the research was conducted in the absence of any commercial or financial relationships that could be construed as a potential conflict of interest.

## Abbreviations

2CPA/2CPB: 2-Cys-Peroxiredoxin A/B
ABA: abscisic acid
Col/Col-0: *Arabidopsis thaliana* var. Columbia-0 wildtype
CAPS: cleaved amplified polymorphic sequence
CPR1: constitutive expresser of PR1
CSD2: copper/zinc superoxide dismutase
DAB, 3: 3′-diaminobenzidine
ECS: γ-glutamyl-cysteine synthase
ETI: effector triggered immunity
GC-MS: gas chromatography coupled to mass spectrometry
GR: glutathione reductase
HL: High light (800 μmol photons m^−2^ s^−1^)
JA: jasmonic acid
JA-Ile: Isoleucine conjugated jasmonic acid
Ler: *Arabidopsis thaliana* var. Landsberg *erecta* wildtype
LHCA: light-harvesting complex of photosystem I
LHCB: light-harvesting complex of photosystem II
MDAR: monodehydroascorbate reductase
MDH: malate dehydrogenase
NBT: nitroblue tetrazolium
NL: normal light (100 μmol photons m^−2^ s^−1^)
PAS: plastid antioxidant system
PCR: polymerase chain reaction
PET: photosynthetic electron transport
PR: pathogenesis related
qRT-PCR: quantitative polymerase chain reaction following reverse transcription of mRNA
*rimb*: redox-imbalanced
ROS: reactive oxygen species
RUBISCO: Ribulose-1,5-bisphosphate carboxylase/oxygenase
s.d.: standard deviation
sAPX: stromal ascorbate peroxidase
SA: salicylic acid
SNP: single nucleotide polymorphism
SSLP: simple sequence length polymorphism
tAPX: thylakoid-bound ascorbate peroxidase
T-DNA: transfer DNA
*wt*: wildtype.
