# Supplementary material for: The Plant Immunity Regulating F-Box Protein CPR1 Supports Plastid Function in Absence of Pathogens
Source: Front Plant Sci. 2017 Sep 22;8:1650. doi: 10.3389/fpls.2017.01650 (PMC5615928; doi:10.3389/fpls.2017.01650)
Supplement: Supplementary file 1 [file Table1.PDF]

Suppl. Tab. 1

The plant immunity regulating F-box protein *CPR1* supports plastid function in absence of pathogens

Christiane Hedtmann[1], Wei Guo[1], Elena Reifschneider[1], Isabelle Heiber[2], Heiko Hiltscher[3], Jörn van Buer[1], Aiko Barsch[4], Karsten Niehaus[4], Beth Rowan[5], Tobias Lortzing[6], Anke Steppuhn[6], Margarete Baier[1*]

List of primers

T-DNA verification

SALK_111420 (cpr1-5)_LP	AACAGACACCGGATATGAACG
SALK_111420 (cpr1-5)_RP	TCGTGGTTACGTGTTTTACGG
SALK T-DNA specific	ATTTTGCCGATTTCGGAAC

Confirmation of the mutation

At4g12560_f	TCGTCGATTCAGCTTTTG
At4g12560_r	ACCCCAAGACAAGGCTAC

Primers used for transcript abundance analysis depicted in Fig. 2, 3, 6, 7 and 11

Annotation	*AGI code*	**forward**	**reverse**
2CPA	At3g11630	CCCAACAGAGATTACTGCCT	ATAGTTCAGATCACCAAGCCC
Actin2	At3g18780	AATCACAGCACTTGCACCAAGC	CCTTGGAGATCCACATCTGCTG
F-Box	At5g15710	TTTCGGCTGAGAGGTTCGAGT	GATTCCAAGACGTAAAGCAGATCAA
MDAR	At1g63940	TGGGAGAAACAGTGGAGGTTGG	TGGTAGAAGCTGGAACTCCTCAG
PAD4	At3g52430	AGATACGCGAGCACAACGCAAG	TTCTCGCCTCATCCAACCACTC
PR1	At2g14610	TTCTTCCCTCGAAAGCTCAA	AAGGCCCACCAGAGTGTATG
PR2	At3g57260	AGCTTCCTTCTTCAACCACACAGC	TGGCAAGGTATCGCCTAGCATC
CPR1	At4g12560	TGTCCAAAGGATTCAAGCTGGTC	TCGTTTCTCCTCCCTTGTGCAG
tAPX	At4g08390	GCTAGTGCCACAGCAATAGAGGAG	TGATCAGCTGGTGAAGGAGGTC
SID2	At3g62030	GCTTGGCTAGCACAGTTACAGC	CACTGCAGACACCTAATTGAGTCC
BAP1	At3g61190	ATCGGATCCCACCAGAGATTACGG	AATCTCGGCCTCCACAAACCAG
ZAT10	At1g27730	TCACAAGGCAAGCCACCGTAAG	TTGTCGCCGACGAGGTTGAATG
YLS8	At5g08290	TTACTGTTTCGGTTGTTCTCCATTT	CACTGAATCATGTTCGAAGCAAGT

Primers used transcript abundance analysis depicted in Fig. 8 and 9

Annotation	*AGI code*	**forward**	**reverse**
Actin7	At5g09810	GAGAAGATGACTCAGATC	ATCCTTCCTGATATCGACATCA
2CPA	At3g11630	CTCTCCATCTGTTTCTTT	GTACCTTTTTGCTATCAT
2CPB	At5g06290	ATAGCTTCTTCTTCTTCC	CATGTGTTCAATCTTAGC
PRXQ	At3g26060	TGGCTCCACACTCACTCA	TCAGGCTGGAACTGGTTG
sAPX	At4g08390	TGTTCCAGTTAGCTAGTG	GGTTGAGTAAATTAGGTGC
tAPX	At1g77490	AATAGTTGCCTTGTCTGG	GGAATATATGATCACCACG
APX1	At1g07890	CTGATTTCCATCAGCTTGCT	TCAGCAGCGTATTTCTCGAC
APX2	At3g09640	ACCCGCTCATTTTTGACAAC	AGCAAACCCGAGTTCTGACA
CAT2	At4g35090	AGCAACTTGCTTTCTGTC	TTAGATGCTTGGTCTCAC
CSD2	At2g28190	CTCCGTTCCTCTTTCAGC	GCGTCAAGCCAATCACAC

Suppl. Tab. 1

MHAR	At1g63940	TTGGAATTGGAGCTAAGC	TTCTTCGACTGAAGATGC
γECS	At4g23100	CCTATGTACTTTGCCTAC	CCTTATTCCGGAGACTCG
GR	At3g54660	TTGCAATAGTTGGTGGTG	TTGCAACCTTCTCTACAC
MDH	At5g58330	ATGAAGAGATCCAAGAGC	CTACTTCAATGCCATGTTC
ApL3	At4g39210	CGATCTGAAATCAATGCAAACC	GCATTTCCTGATCTTTGTATCTCG
STP1	At1g11260	TTCTTTCAACAGCTAACCGGAATCA	GGCTAATACACTTTTTCCTTTACGACA
RBCS	At5g38430	AGAAGTAATGGCTTCCTC	AAGCTTCGGTGAAGCTTG
PETC	At4g03280	TACCTTCTTTGTTCCTCCTG	AGAGTCTTAAGACCACCATG
PETM	At2g26500	TCAGCAAGGAATGGCGAC	AACGAATCCAACTGCAACAC
PETE1	At1g76100	TTCTCTTGTTCGTGTAGGAGCTT	TCGGATTGTATGACCGTGAC
PETE2	At1g20340	TGTGTGTGAGTAAGTGAGGGAGT	CCATTTATGGAACCAGTCACAG
LHCA5	At1g45474	GGAGATTACGGGTTTGAC	TGATGGTCTTATGCCATG
LHCB2.2	At2g05070	CGTCTTACTTAACTGGAG	TTAGTAGCGTAAGACCAG
LHCB4.1	At5g01530	CGGAATCCAGAGATTCAG	CCCAATTGTTGAGTGGAC
BAP1	At3g61190	CTAAACCGGAGACCCATC	AGTGACCTTCAGGTGAATAC
FER1	At5g01600	ATGGCCTCAAACGCACTCTCGTC	CTAGTCCCTTCATAGCAACG
LOX2	At3g45140	CCTGATGAAGAACTGATC	AAGAGACAGAGATACAG
