# Supplementary material for: The Plant Immunity Regulating F-Box Protein CPR1 Supports Plastid Function in Absence of Pathogens
Source: Front Plant Sci. 2017 Sep 22;8:1650. doi: 10.3389/fpls.2017.01650 (PMC5615928; doi:10.3389/fpls.2017.01650)
Supplement: Supplementary file 2 [file Table2.PDF]

Suppl. Tab. 2

The plant immunity regulating F-box protein *CPR1* supports plastid function in absence of pathogens

Christiane Hedtmann[1], Wei Guo[1], Elena Reifschneider[1], Isabelle Heiber[2], Heiko Hiltscher[3], Jörn van Buer[1], Aiko Barsch[4], Karsten Niehaus[4], Beth Rowan[5], Tobias Lortzing[6], Anke Steppuhn[6], Margarete Baier[1*]

SNPs between *rimb6 (cpr1-4)* and T19-2 on the top arm of chromosome IV

Position	Reference base	Alternate base	Gene	Codon position in gene	type	Reference AA	New AA
**7078331**	G	A	AT4G11750	220	Nonsyn	R	C
**7442672**	G	A	AT4G12560	858	Nonsyn	W	*
**8219957**	A	-	AT4G14272	214	Nonsyn	K	X
**8699123**	T	A	AT4G15236	1621	Nonsyn	L	M
**8974535**	C	-	AT4G15760	14	Nonsyn	G	X
**8975844**	C	-	AT4G15765	755	Nonsyn	G	X
**8976974**	A	-	AT4G15765	136	Nonsyn	W	X
**8980212**	G	-	AT4G15780	540	Nonsyn	T	X
**8980214**	T	-	AT4G15780	538	Nonsyn	T	X
**8989799**	C	-	AT4G15810	2060	Nonsyn	G	X
**8993849**	T	-	AT4G15820	794	Nonsyn	V	X
**8126091**	G	A	AT4G14096	1296	Syn	R	R
