# Supplementary material for: The Plant Immunity Regulating F-Box Protein CPR1 Supports Plastid Function in Absence of Pathogens
Source: Front Plant Sci. 2017 Sep 22;8:1650. doi: 10.3389/fpls.2017.01650 (PMC5615928; doi:10.3389/fpls.2017.01650)

Suppl. Fig. 3

The plant immunity regulating F-box protein *CPR1* supports plastid function in absence of pathogens

Christiane Hedtmann[1], Wei Guo[1], Elena Reifschneider[1], Isabelle Heiber[2], Heiko Hiltscher[3], Jörn van Buer[1], Aiko Barsch[4], Karsten Niehaus[4], Beth Rowan[5], Tobias Lortzing[6], Anke Steppuhn[6], Margarete Baier[1*]

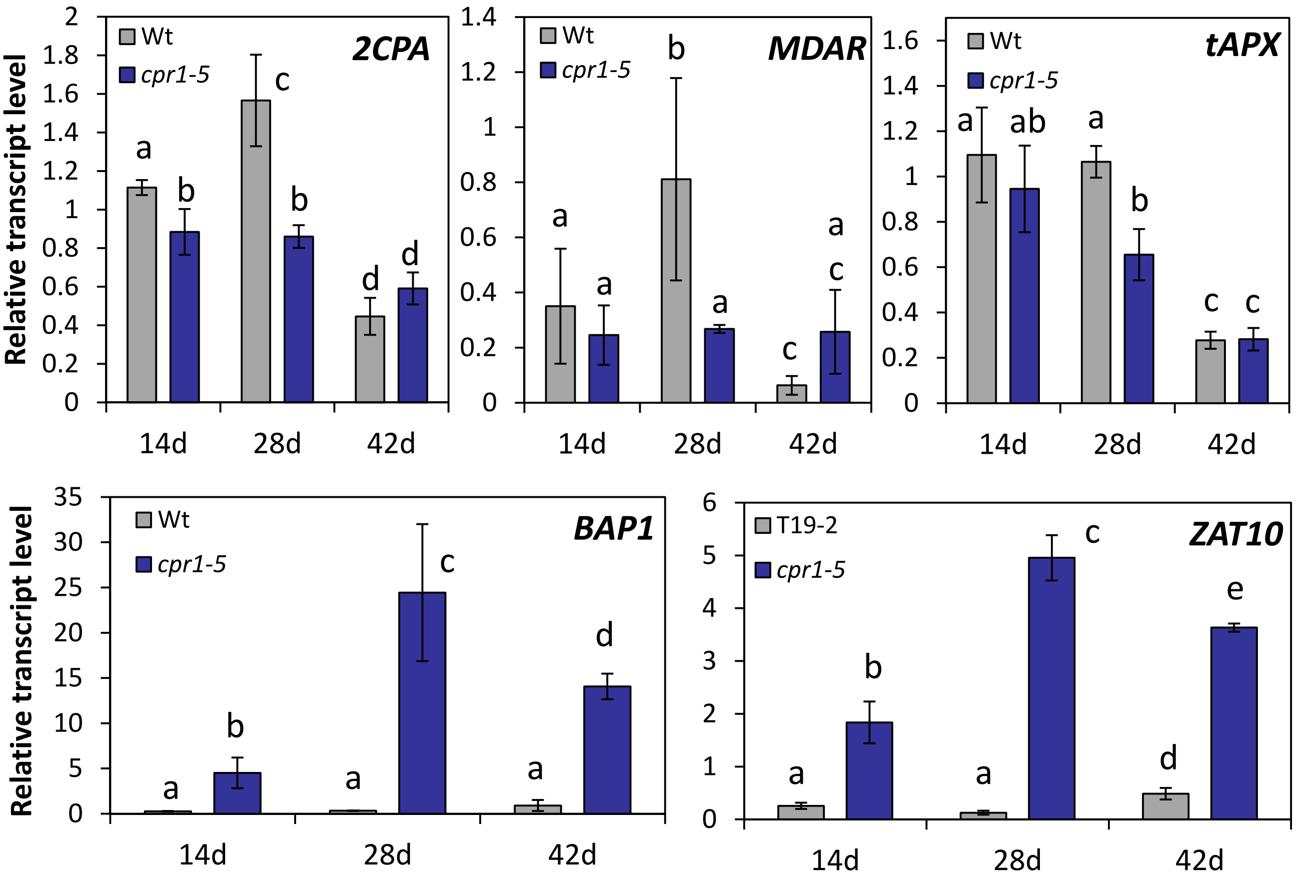

Supplement: Supplementary file 5 [file SupplementaryFigure3.PDF]
